# G protein α subunit suppresses sporangium formation through a serine/threonine protein kinase in *Phytophthora sojae*

**DOI:** 10.1371/journal.ppat.1008138

**Published:** 2020-01-21

**Authors:** Min Qiu, Yaning Li, Xin Zhang, Mingrun Xuan, Baiyu Zhang, Wenwu Ye, Xiaobo Zheng, Francine Govers, Yuanchao Wang

**Affiliations:** 1 Department of Plant Pathology, Nanjing Agricultural University, Nanjing, Jiangsu, China; 2 The Key Laboratory of Integrated Management of Crop Diseases and Pests (Ministry of Education), Nanjing, Jiangsu, China; 3 Laboratory of Phytopathology, Wageningen University, Wageningen, The Netherlands; Institute of Microbiology, CHINA

## Abstract

Eukaryotic heterotrimeric guanine nucleotide-binding proteins consist of α, β, and γ subunits, which act as molecular switches to regulate a number of fundamental cellular processes. In the oomycete pathogen *Phytophthora sojae*, the sole G protein α subunit (Gα; encoded by *PsGPA1*) has been found to be involved in zoospore mobility and virulence, but how it functions remains unclear. In this study, we show that the Gα subunit PsGPA1 directly interacts with PsYPK1, a serine/threonine protein kinase that consists of an N-terminal region with unknown function and a C-terminal region with a conserved catalytic kinase domain. We generated knockout and knockout-complemented strains of *PsYPK1* and found that deletion of *PsYPK1* resulted in a pronounced reduction in the production of sporangia and oospores, in mycelial growth on nutrient poor medium, and in virulence. PsYPK1 exhibits a cytoplasmic-nuclear localization pattern that is essential for sporangium formation and virulence of *P*. *sojae*. Interestingly, *PsGPA1* overexpression was found to prevent nuclear localization of PsYPK1 by exclusively binding to the N-terminal region of PsYPK1, therefore accounting for its negative role in sporangium formation. Our data demonstrate that PsGPA1 negatively regulates sporangium formation by repressing the nuclear localization of its downstream kinase PsYPK1.

## Author summary

Eukaryotic heterotrimeric guanine nucleotide-binding proteins consist of α, β, and γ subunits, which act as molecular switches to regulate a number of fundamental cellular processes. In oomycete plant pathogen *Phytophthora sojae*, previous research showed that G protein α subunit is involved in zoospore motility and pathogenicity. However, the elaborate functions of the G protein α subunit remain largely unknown. Here, we identified a serine/threonine protein kinase, PsYPK1, which directly interacts with the G protein α subunit (PsGPA1) in *P*. *sojae*. We found that *PsYPK1* regulates the production of sporangia and oospores, mycelial growth in nutrient poor medium, and pathogenicity in *P*. *sojae*. We further revealed that PsYPK1 exhibits a cytoplasmic-nuclear localization pattern that is essential for sporangium formation and pathogenicity of *P*. *sojae*. Interestingly, *PsGPA1* overexpression was found to prevent nuclear localization of PsYPK1 by exclusively binding to the N-terminal region of PsYPK1, accounting for the negative role of PsGPA1 in sporangium formation. Our study is the first to show that G protein signaling is transmitted to a downstream effector to regulate the development and pathogenicity of an oomycete pathogen, which opens new avenues for researches on animal and plant pathogens and host-microbe interactions.

## Introduction

*Phytophthora* species are destructive plant pathogens that attack a wide range of ornamentally and agriculturally important plants. Although *Phytophthora* species exhibit a fungus-like filamentous growth morphology, they are distant relatives to fungi in evolution. The genus *Phytophthora* belongs to the oomycetes, a lineage in the *Stramenopila* kingdom, which constitutes a distinct, major branch in the eukaryotic evolutionary tree and comprises saprophytes and pathogens of plants, animals, and insects [1, 2]. *Phytophthora sojae* is one of the many plant pathogen oomycetes and the causal agent of root and stem rot in soybean. It is distributed throughout most soybean-growing regions, and causes plant stand reductions and even complete yield losses with damage valued at approximately $1–2 billion each year [3, 4]. In addition to an abundance of available genome and transcriptome data [1, 5], the recently established clustered regularly interspaced short palindromic repeats (CRISPR)-mediated gene knockout system has greatly strengthened functional genomic research in *P*. *sojae* [6, 7].

Heterotrimeric G proteins are key regulators for transducing extracellular signals to intracellular signaling pathways and are essential for viability of many eukaryotes [8]. In animals and fungi, the G protein complex comprising a Gα, Gβ, and Gγ subunit is activated by G protein-coupled receptors (GPCRs) upon binding of an extracellular ligand. GTP binding causes a conformational change in the Gα subunit thereby disrupting the interaction of Gα with the Gβγ dimer. The dissociated and therefore activated Gβγ dimer and Gα GTP subunit are now able to interact with their target proteins, which in the context of G protein signaling, are also referred to as effectors [9]. The human genome encodes 23 Gα, five Gβ, and 12 Gγ subunits. Downstream effectors of activated Gα and Gβγ subunits in animals are, amongst others, protein kinase C, adenylate cyclase, and ion channels [10]. Most filamentous fungi in which G proteins have been studied possess three Gα proteins that are associated with nutrient sensing, pheromone responses, chemotaxis, mating, and pathogenesis [8, 11, 12]. Compared to animals, plants have a reduced repertoire of G proteins with usually one Gα subunit, one Gβ subunit, and three to five Gγ subunits [13–15]. Although the three-dimensional structure of the *Arabidopsis* Gα protein is nearly identical to the structure of human Gα proteins [16], plant Gα spontaneously exchanges GDP for GTP without any involvement of GPCRs, and is thus proposed to be constitutively active [17, 18]. In *Arabidopsis*, the G protein pathway acts as a molecular rheostat to modulate a multitude of other signaling pathways, including nutrient sensing, light sensitivity, hook opening, anthocyanin synthesis, abscisic acid regulation of seed germination, and post-germination development [19–22].

It is well established that heterotrimeric G proteins can regulate downstream effectors through a variety of mechanisms. For oomycetes, however, little is known about the molecular players involved in heterotrimeric G protein signaling. *Phytophthora* spp. have only one Gα, one Gβ, and one Gγ subunit [23, 24]. In the potato late blight pathogen *Phytophthora infestans* the Gα subunit PiGPA1 has roles in swimming behavior and chemotaxis of zoospores, whereas Gβ and Gγ (PiGPB1 and PiGPG1, respectively) are critical for sporangia development and zoosporogenesis [23, 25, 26]. Also in *P*. *sojae* the single Gα subunit protein PsGPA1 is important for zoospore functioning because silencing of the encoding gene leads to defects in zoospore chemotaxis and encystment [27]. Attempts to find proteins interacting with PsGPA1 resulted in the identification of a histidine triad nucleotide-binding protein 1 (PsHint1) that is also involved in zoospore chemotaxis, and required for cyst germination and full virulence [28]. Apart from PsHint1, however, the downstream effectors or pathways mediated by *Phytophthora* G proteins and the signals recognized by the single G protein complex are largely unknown.

In this study, we identified a serine-threonine kinase in *P*. *sojae*, called PsYPK1, which directly interacts with the Gα protein PsGPA1. We found that PsGPA1 acts as a negative regulator of sporangium formation by inhibiting the nuclear localization of PsYPK1. Furthermore, PsYPK1 has Gα-independent pathways that are involved in mycelium growth and oospore formation. Our data support a model in which the G protein α subunit suppresses sporangium formation in *P*. *sojae* through a serine/threonine protein kinase.

## Results

### The PsGPA1 protein interacts with the N-terminal region of PsYPK1

**Fig 1:**
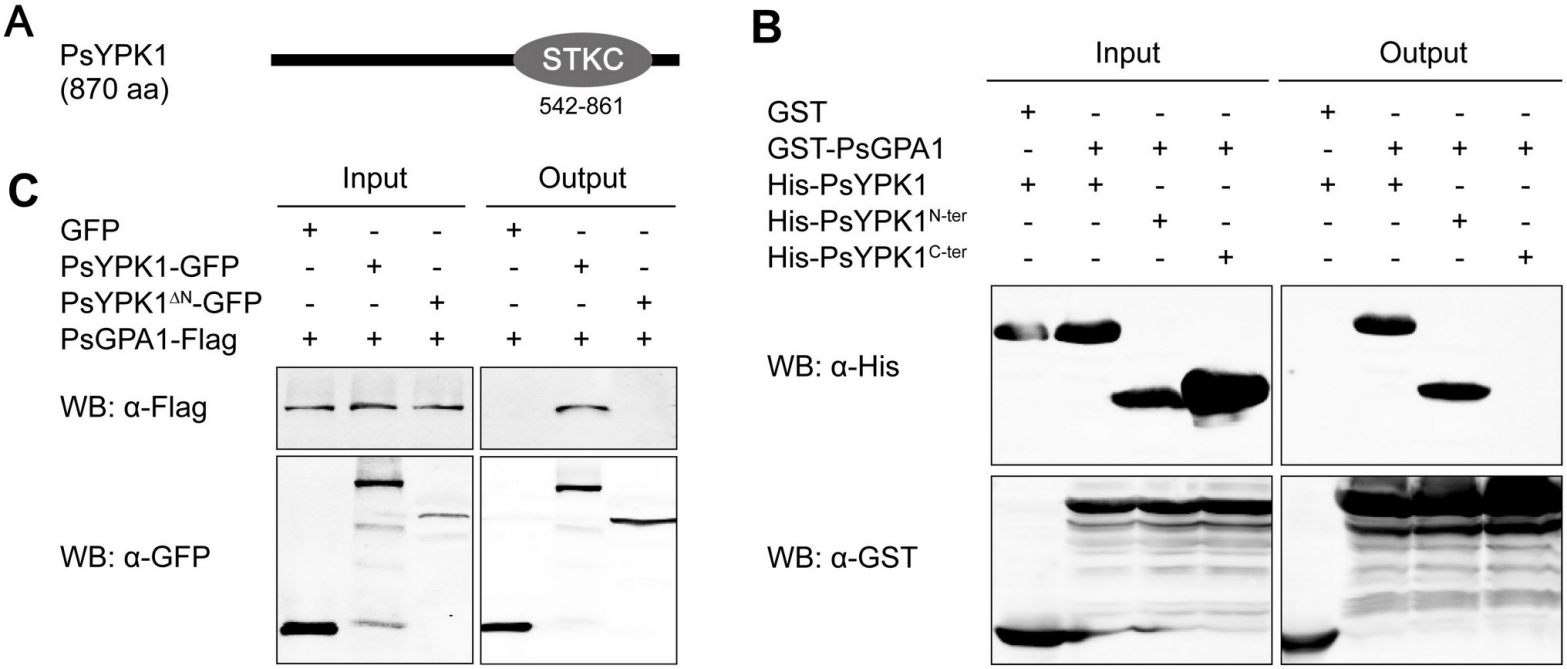
PsGPA1 physically interacts with the N-terminal region of PsYPK1 *in vitro* and *in vivo*. (A) The domain architecture of PsYPK1. The numbers under the STKc (serine/threonine protein kinases, catalytic) domain are the amino acid positions in the full-length protein. (B) Confirmation of the association of PsGPA1 and PsYPK1 *in vitro*. GST-PsGPA1- or GST-bound resins were incubated with *E*. *coli* supernatant containing His-PsYPK1, or the N-terminal or the C-terminal portions of PsYPK1. The presence of His-tagged proteins was detected by western blot analysis using a His antibody. (C) Validation of the association between PsYPK1 and PsGPA1 *in vivo*. Co-immunoprecipitations (Co-IP) were performed in extracts of *Phytophthora sojae* mycelium expressing PsGPA1-FLAG with PsYPK1-GFP or PsYPK1^ΔN^-GFP. The presence of FLAG-tagged proteins was detected by western blot analysis using a FLAG antibody. The bands detected with anti-GFP were quantified with the ODYSSEY infrared imaging system (application software version 2.1).

To identify PsGPA1-interacting proteins, we obtained a *P*. *sojae* transformant expressing 3×FLAG-tagged PsGPA1 and performed protein co-immunoprecipitation (Co-IP) assays on lysates of this transformant using FLAG antibodies as bait (**S1A Fig**). Mass spectroscopy (MS) analysis of the complex co-precipitating with PsGPA1 identified 65 candidates as potential interactors of PsGPA1 including two serine/threonine kinases (i.e. Ps350990 and Ps350499) for which the interaction with PsGPA1 was confirmed (**S1 Table and S1B Fig**). Phylogenetic analysis showed that Ps350990 is one of several protein kinases in *P*. *sojae* that are related to *Saccharomyces cerevisae* ScYPK1/2 (**S2A Fig**). Although Ps350990 is not the closest homologue of ScYPK1/2 in *P*. *sojae*, it is the only one that is conserved with ScYPK1 at all key positions for phosphorylation and kinase activity (**S2B Fig**) and hence we named it PsYPK1. PsYPK1 is 870 amino acids in size and apart from a conserved serine/threonine catalytic domain (amino acids 542–861) in the C-terminal part it has no known domains (**Fig 1A**). It belongs to the AGC family of serine/threonine kinases, one of the most evolutionarily conserved groups of protein kinases in eukaryotes [29]. Based on BLAST searches using the PsYPK1 protein sequence as query, homologs were identified in other *Phytophthora* spp. Sequence alignment with homologs from organisms in the various kingdoms revealed that the proteins have a conserved C-terminal catalytic domain preceded by a highly variable N-terminal region that is relatively large in *Phytophthora* YPK1 proteins (**S3 Fig**).

To verify the protein-protein interaction between PsGPA1 and PsYPK1 we used an *in vitro* glutathione S-transferase (GST) pull-down assay (**Fig 1B**). GST-PsGPA1 and 6×His-PsYPK1 were expressed separately in *Escherichia coli*, and then purified for use in a pull-down experiment. As shown in **Fig 1B**, 6×His-PsYPK1 bound specifically to GST-PsGPA1, but not to GST, suggesting that the two proteins interact directly. To confirm that this interaction also occurs *in vivo*, PsGPA1-FLAG was co-expressed with PsYPK1-GFP in *P*. *sojae* and lysates of the mycelium were used in Co-IP assays. Also these assays showed a specific interaction between the two proteins (**Fig 1C**). To determine which region of PsYPK1 is responsible for the PsGPA1-PsYPK1 interaction, deletion constructs of PsYPK1 were generated and tested in the interaction assays. The results revealed that deletion of the N-terminal region of PsYPK1 (N-ter, amino acids 2–303) abolished its interaction with PsGPA1, both in the *in vitro* (**Fig 1B**; His-PsYPK1^C-ter^) and *in vivo* assay (**Fig 1C**; PsYPK1^ΔN^-GFP). In contrast, a construct lacking amino acids 543–861 in the C-terminal region retained its ability to interact with PsGPA1 (**Fig 1B**; His-PsYPK1^N-ter^). These data suggest that PsYPK1 directly associates with PsGPA1 and that its N-terminal region is required and sufficient for the interaction with PsGPA1.

### PsYPK1 is required for growth of *P*. *sojae* in nutrient poor conditions and for oospore production, sporangium formation and virulence

To investigate the biological function of PsYPK1 we generated *PsYPK1* knockout mutants using CRISPR-mediated gene replacement [6] as well as complemented knockout mutants, and analyzed their phenotypes. We show the results obtained from one representative *PsYPK1* knockout mutant, ΔPsYPK1, and two representative *PsYPK1* complemented strains, ΔPsYPK1-C1 and ΔPsYPK1-C2. The recipient wild-type strain (P6497) and two empty vector lines (EV in which the *PsYPK1* knockout was not successful and ΔPsYPK1-EV that was not successfully complemented with *PsYPK1*) were included as controls (**S4A–S4D Fig**).

**Fig 2:**
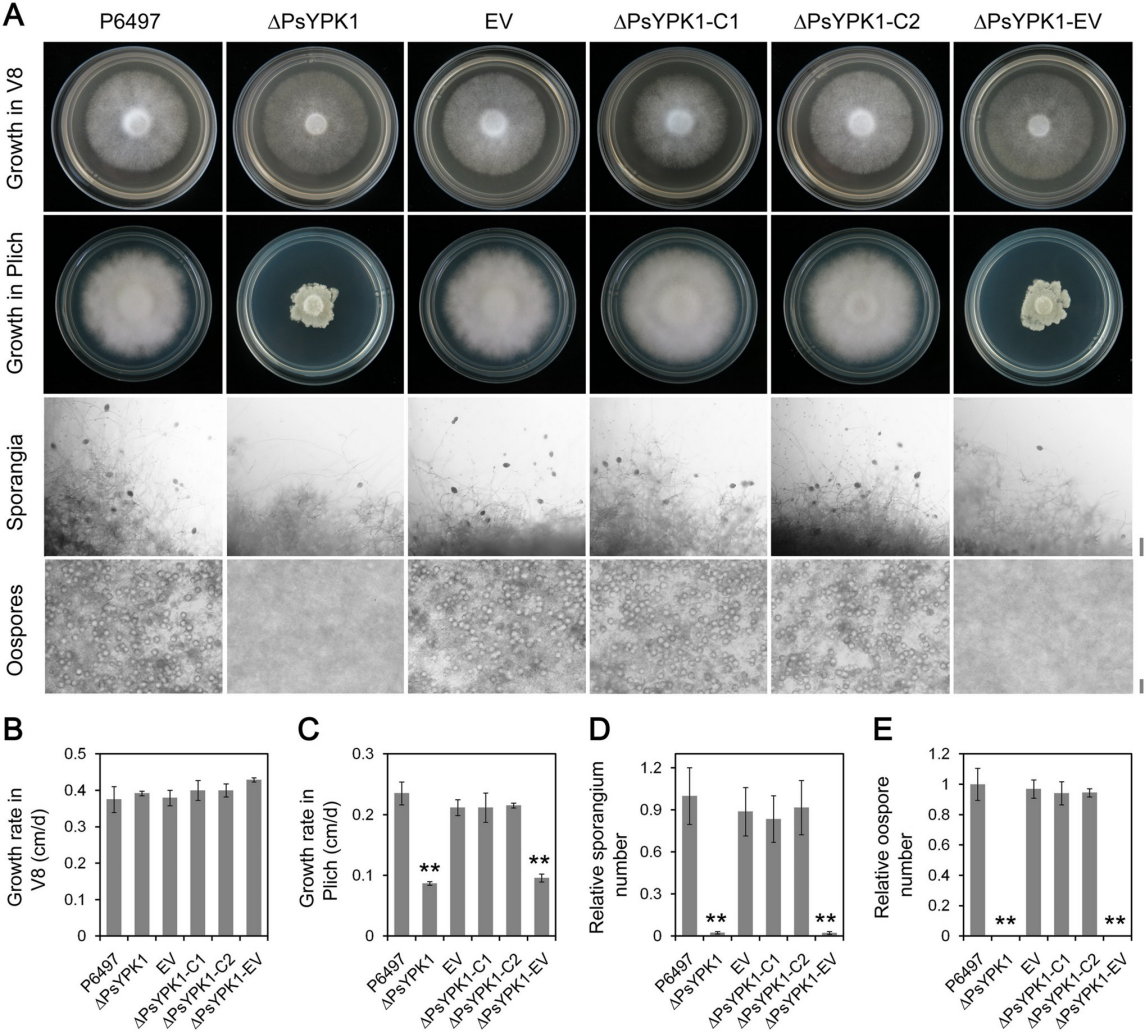
PsYPK1 is important for growth in nutrient poor conditions and for sporangium and oospore production. (A) Growth characteristics after 4 days on V8 medium and 7 days on Plich medium, and microscopic visualization of oospores and sporangia of the wild-type (P6497), PsYPK1-knockout (ΔPsYPK1), empty vector control line (EV), complemented transformants (ΔPsYPK1-C1, C2), and empty control line of ΔPsYPK1 (ΔPsYPK1-EV). (B) Growth rates in cm/day on V8 medium and (C) Plich medium. (D) The relative numbers of sporangia and (E) oospores with the number in wild-type strain P6497 set at 1. All experiments were repeated three times with similar results. Scale bar, 50 μm. Asterisk indicates significant difference at *P<0*.*01* (**).

When cultured on nutrient-rich V8 medium no significant differences in growth characteristics were observed (**Fig 2A and 2B**), whereas on nutrient-poor Plich medium a clear reduction in growth was observed for ΔPsYPK1 and ΔPsYPK1-EV (**Fig 2A and 2C**). When substituting glucose, the major nutrient source in Plich medium, by other monosaccharides we observed that in the presence of fructose ΔPsYPK1 and ΔPsYPK1-EV also showed a decreased growth rate while in the presence xylose, galactose and mannose the growth rate of the mutants was comparable to that of the wild-type strain P6497 (**S5 Fig**). With respect to sporangium formation P6497, EV, ΔPsYPK1-C1, and ΔPsYPK1-C2 all formed abundant sporangia, while ΔPsYPK1 and ΔPsYPK1-EV exhibited a significant reduction (97%) in sporangium number (**Fig 2A and 2D**). Moreover, ΔPsYPK1 and ΔPsYPK1-EV lost the ability to produce oospores (**Fig 2A and 2E**).

**Fig 3:**
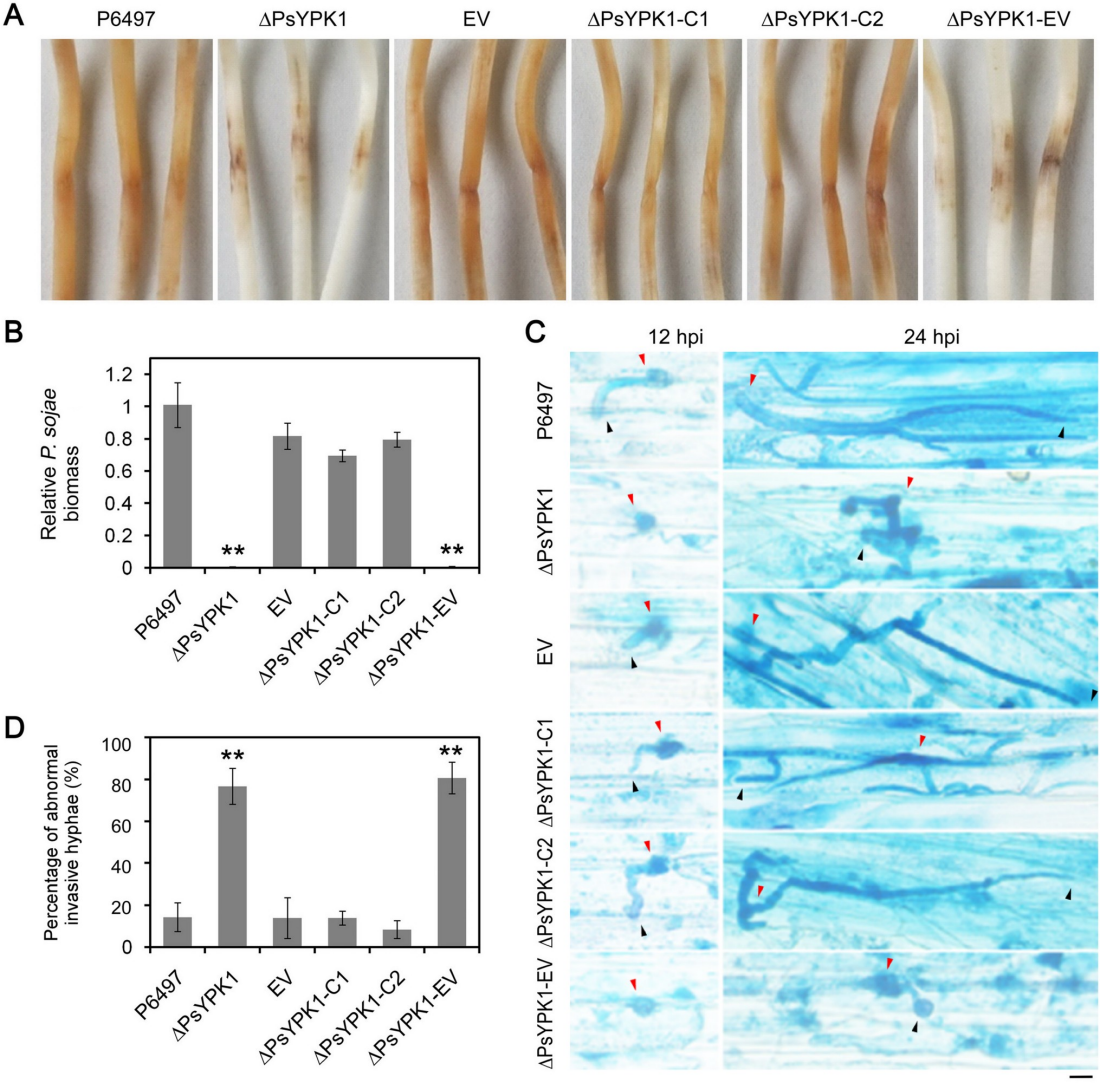
The PsYPK1 knockout mutant is significantly impaired in virulence. (A) Lesions on soybean (cultivar Hefeng 47) at 72 hours post inoculation (hpi) of 4-day-old etiolated hypocotyls with zoospores of the wild-type strain (P6497), PsYPK1-knockout (ΔPsYPK1), empty vector control line (EV), complemented transformants (ΔPsYPK1-C1, C2), and empty control line of ΔPsYPK1 (ΔPsYPK1-EV). Experiments were repeated three times with similar results. (B) Relative pathogen biomass in inoculated etiolated hypocotyls expressed as the ratio between the amounts of *P*. *sojae* DNA and soybean DNA detected at 72 hpi with the ratio P6497/soybean set at 1. Asterisks indicate significant differences at *P<0*.*01* (**). (C) Microscopic observations of invasive hyphae in epidermis of soybean hypocotyls at 12 hpi and 24 hpi. Trypan Blue staining was performed on the epidermis of seedling hypocotyls. Red arrowheads, cysts; black arrowheads, invasive hyphae. Bar, 10 μm. (D) Percentage of abnormal invasive hyphae in epidermal cells of soybean hypocotyls at 24 hpi. In each sample 50 invasive hyphae were examined and the experiments were repeated three times.

When *P*. *sojae* invades and colonizes soybean the transcription of *PsYPK1* is highly upregulated suggesting a role for PsYPK1 during infection stages (**S6A Fig**). Soybean seedlings inoculated with ΔPsYPK1 or ΔPsYPK1-EV developed only small necrotic lesions at the site of inoculation, whereas P6497, EV, ΔPsYPK1-C1, and ΔPsYPK1-C2 all produced normal disease lesions (**Fig 3A**). Measurements of relative *P*. *sojae* biomass in infected soybean seedlings revealed hardly any pathogen biomass in ΔPsYPK1- or ΔPsYPK1-EV-infected tissues with levels less than 1% of the levels found in P6497-, EV-, ΔPsYPK1-C1-, or ΔPsYPK1-C2-infected tissues (**Fig 3B**).

To explore why the pathogenicity of the ΔPsYPK1 strain was impeded, epidermal cells from the seedling inoculation sites were excised at different time points and observed using microscopy. At 12 h post-inoculation (hpi), P6497, EV, ΔPsYPK1-C1, and ΔPsYPK1-C2 were able to penetrate into the epidermal cells and the invasive hyphae were able to expand into adjacent epidermal cells, whereas the hyphae of ΔPsYPK1 and ΔPsYPK1-EV were restricted to the regions surrounding the penetration site (**Fig 3C**). At 24 hpi, the infectious hyphae of the P6497, EV, and the complemented strains exhibited tapering tips and were much more abundant, which had extended into neighboring cells (**Fig 3C**). Approximately 76% of the infectious hypha produced by ΔPsYPK1 or ΔPsYPK1-EV showed abnormal growth in branches and apical swelling, which may contribute to the reduction in the number of successful *P*. *sojae* infections (**Fig 3C and 3D**). Taken together these results indicate that PsYPK1 has a role in growth in nutrient poor medium, oospore production, sporangium formation, and virulence.

### PsGPA1 is a negative regulator of sporangium formation in *P*. *sojae*

**Fig 4:**
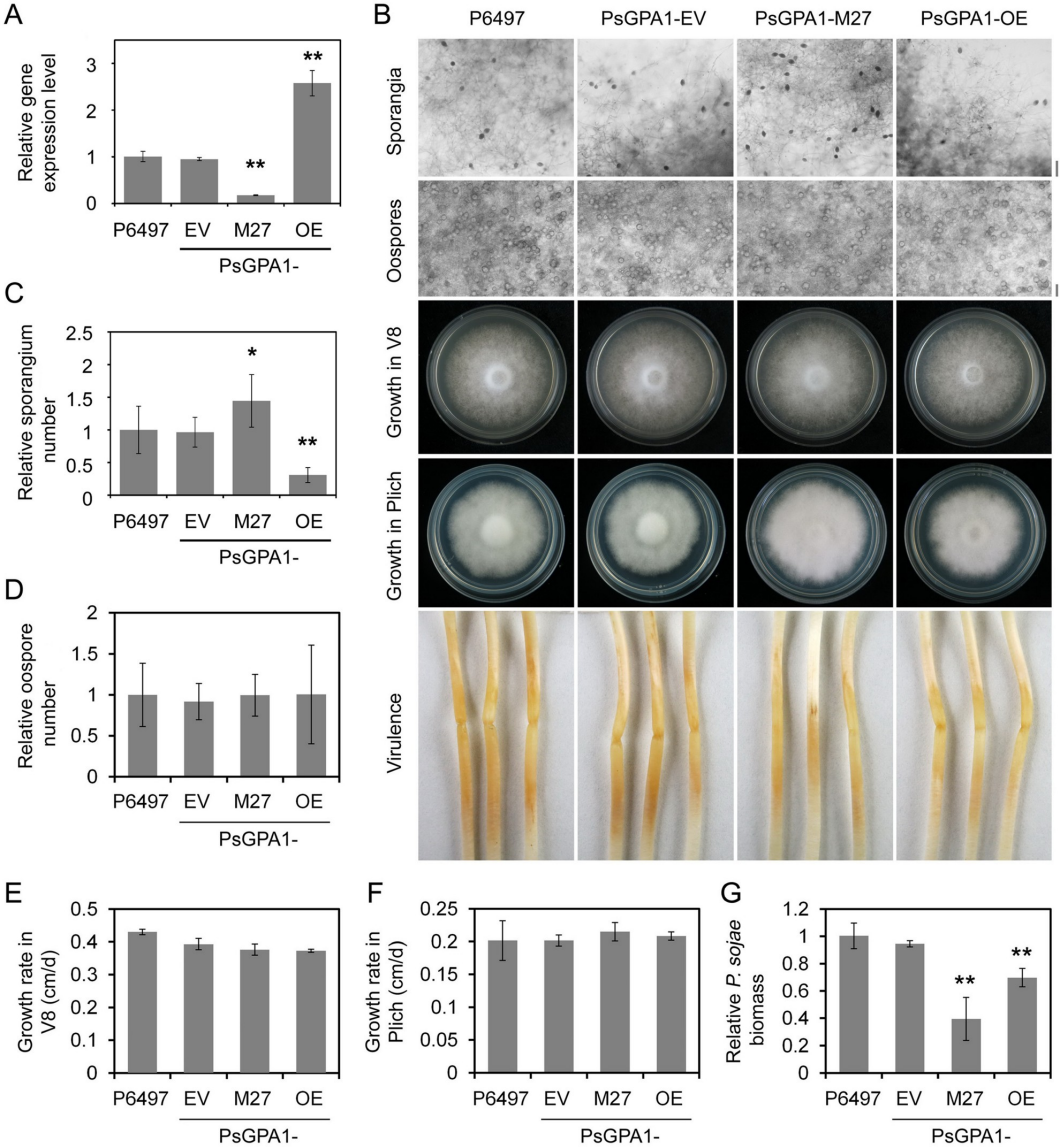
PsGPA1 negatively regulates sporangium formation in *P*. *sojae*. (A) Expression of *PsGPA1* in hyphae of *PsGPA1* silenced and overexpression strains analysed by qRT-PCR). Relative expression levels of the empty control line (PsGPA1-EV), the *PsGPA1*-silenced mutant (PsGPA1-M27), and the *PsGPA1* overexpression strain (PsGPA1-OE) were calculated using the expression level of the wild-type strain P6497 as reference. All qRT-PCR experiments were repeated three times using independent RNA isolations. (B) Microscopic visualization of oospores and sporangia, growth characteristics after 4 days on V8 medium and 7 days on Plich medium, and virulence on soybean hypocotyls of P6497, PsGPA1-EV, PsGPA1-M27, and PsGPA1-OE. (C–G) Quantification of oospore and sporangia production, growth rate and pathogen biomass. All experiments were repeated three times with similar results. Asterisks indicate significant differences at *P<0*.*01* (**), *P<0*.*05* (*).

The high expression levels of the Gα subunit gene *PsGPA1* during sporangium formation could point to a role for Gα in regulating sporangium formation (**S6B Fig**). To test this we generated *PsGPA1-*overexpressing lines using a constitutively active *Ham34* promoter and analyzed the phenotype of one representative line, PsGPA1-OE, which showed a 2.5-fold increase in *PsGPA1* transcript levels compared to the wild-type strain (P6497) and an empty vector control (PsGPA1-EV) (**Fig 4A**). In this analyses we included, a *PsGPA1*-silenced mutant (PsGPA1-M27) generated previously [27] in which the *PsGPA1* transcript levels are strongly reduced (>80%) (**Fig 4A**). PsGPA1-OE exhibited a dramatic reduction (70%) in sporangia number, while the number of sporangia in PsGPA1-M27 was conversely increased (50%; **Fig 4B and 4C**). No obvious changes were found in oospore production and mycelium growth (**Fig 4B and 4D–4F**), indicating that PsYPK1 may have a PsGPA1-independent pathway that regulates vegetative growth and oospore production. Collectively, these results confirm that the Gα protein in *P*. *sojae* plays a negative role during sporangia development. Similar to silencing of *PsGPA1*, *PsGPA1* overexpression also leads to reduced virulence (**Fig 4B and 4G**). This is a confirmation of findings of Hua et al. [27] who showed pleiotropic effects of *PsGPA1-*silencing. Likely the *PsGPA1-*overexpression and silencing causes aberrations in the various pathways regulated by Gα, with several of these governing virulence functions. Moreover, PsGPA1 mutants only showed obvious reduction in virulence on unwounded plants upon inoculation with zoospores but not on wounded plants or upon inoculation with hyphae while PsYPK1 mutant exhibits reduced virulence under all infection conditions **(S6C and S6D Fig)**, indicating that PsYPK1 and PsGPA1 affect the virulence of the pathogen at different levels.

### Nuclear localization of PsYPK1 is determined by the N-terminal region and facilitates sporangium formation

**Fig 5:**
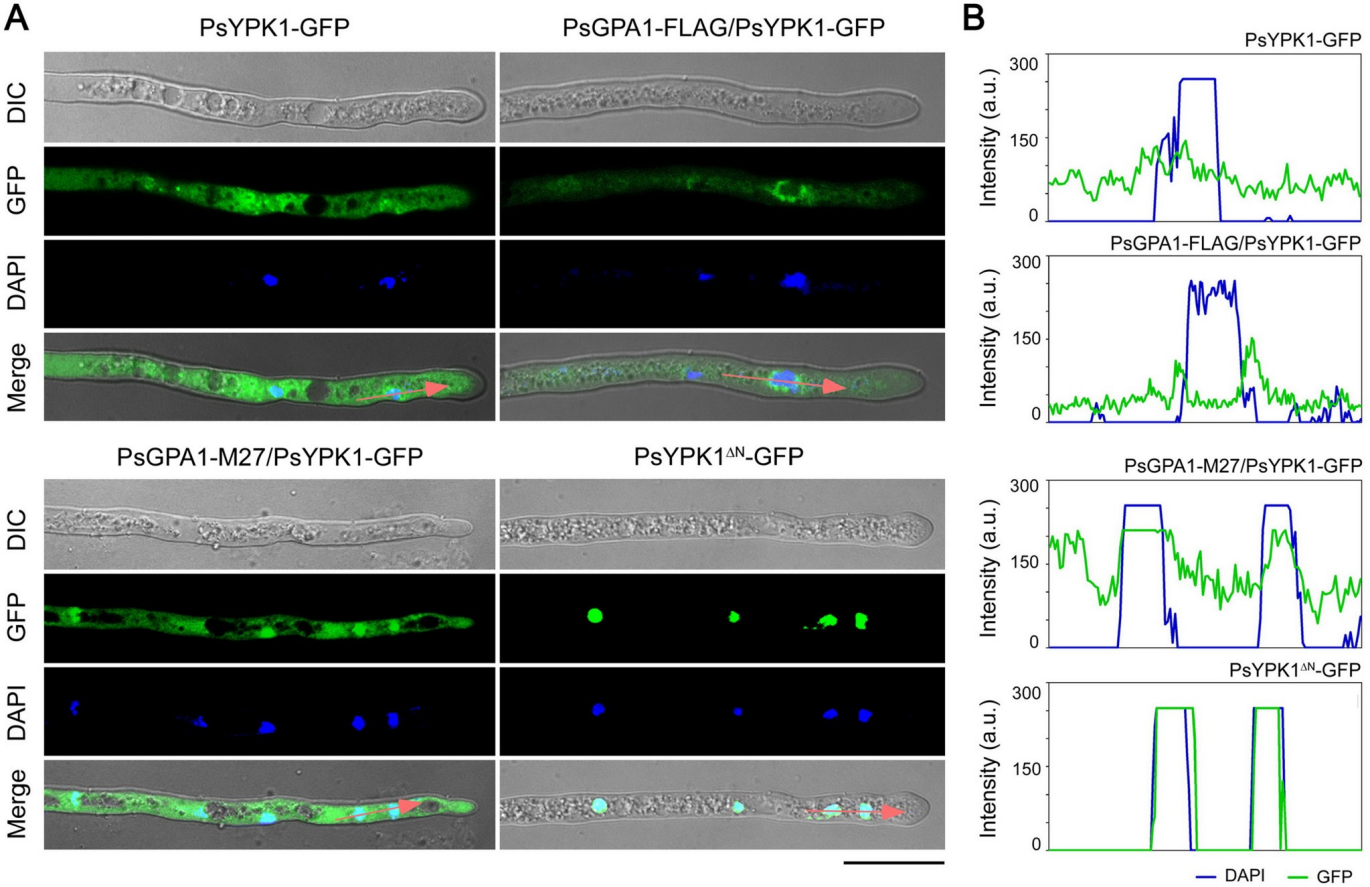
*PsGPA1* overexpression inhibits the nuclear localization of PsYPK1. (A) Microscopic analyses of *P*. *sojae* transformants expressing PsYPK1-GFP or PsYPK1^***ΔN***^–GFP in the wild-type recipient stain P6497 (marked as PsYPK1-GFP or PsYPK1^***ΔN***^–GFP, respectively) or in a *PsGPA1* silenced or overexpression strain (marked as PsGPA1-M27/PsYPK1-GFP and PsGPA1-FLAG/PsYPK1-GFP, respectively). DAPI (4', 6-diamidino-2-phenylindole) staining was performed by adding DAPI to the cultures 5 min prior to the microscopic analysis. DIC: differential interference contrast; Merge: overlay of DIC, GFP fluorescence and DAPI staining. Bar, 20 μm. (B) Relative fluorescence intensity along the red arrows in A. Green line: PsYPK1-GFP or PsYPK1^***ΔN***^–GFP, blue line: nucleus (stained with DAPI).

Given that the N-terminal region of PsYPK1 is essential for its interaction with PsGPA1 (**Fig 1B and 1C**), and that all *Phytophthora* YPK1 homologs have an extended N-terminus (**S3 Fig**) we sought to investigate the role of the N-terminal region. To this end constructs were made in which GFP is fused to either the N-terminal region of *PsYPK1* (PsYPK1^N-ter^-GFP) or to a N-terminal truncated version of *PsYPK1* in which the amino acids 2–303 were deleted (PsYPK1^ΔN^-GFP) and these were transformed into *P*. *sojae*. Hyphae of the resulting transformants and of transformants expressing the full-length *PsYPK1* fused to GFP (PsYPK1-GFP) were examined by fluorescence microscopy (**Fig 5 and S7 Fig**). In the PsYPK1-GFP and PsYPK1^N-ter^-GFP transformants the GFP fluorescence was primarily localized in the cytoplasm, while in the PsYPK1^ΔN^-GFP transformants there was hardly any fluorescence in the cytoplasm. In contrast, the nuclei, visualized by DAPI staining, showed a very strong fluorescence indicating that the truncated PsYPK1^ΔN^-GFP protein accumulates in the nucleus (**Fig 5 and S7 Fig**). Because of the interaction between PsGPA1 and N-terminal region of PsYPK1, the question was raised where PsGPA1 is localized. Fluorescence microscopy of *PsGPA1-GFP* expressing transformants revealed that the fluorescence was mainly visible in cytoplasm and this is in line with the idea that PsGPA1 and PsYPK1 are co-localized in the same subcellular compartment (**S7A–S7C Fig**).

**Fig 6:**
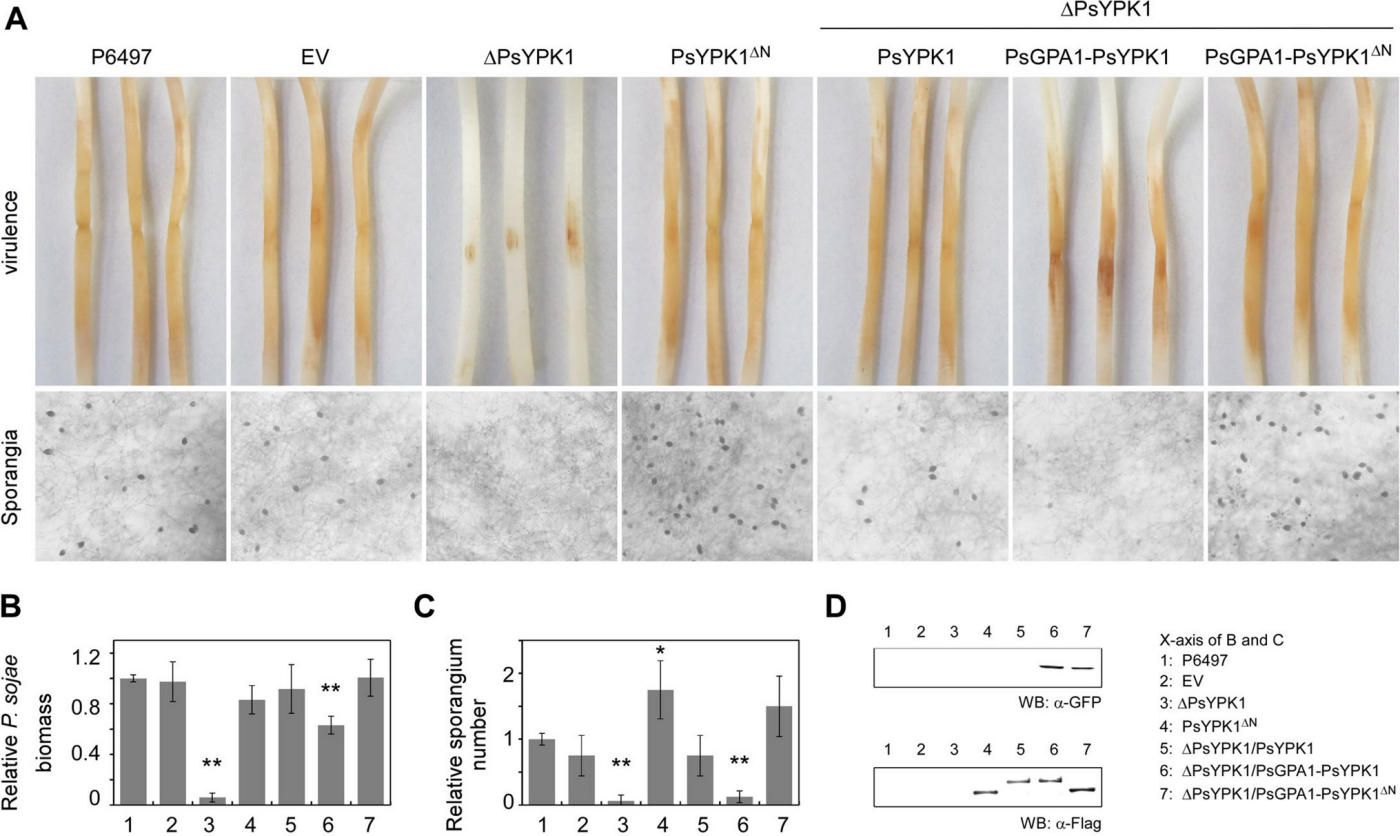
The N-terminal region of PsYPK1 is required for PsGPA1 to suppress sporangium formation. (A) Virulence and sporangium production of wild-type *P*. *sojae* (P6497), and its transformants EV (empty vector control), ΔPsYPK1 (PsYPK1-knockout mutant) and PsYPK1^ΔN^ (*PsYPK1* N-terminal knockout), and of *P*. *sojae* transformants in a ΔPsYPK1 background expressing *PsYPK1* (complemented strain), *PsYPK1* with *PsGPA1*, or *PsYPK1*^*ΔN*^ with *PsGPA1*. For the virulence assays 4-day-old etiolated soybean hypocotyls (cultivar Hefeng 47) were inoculated with zoospores and photographs of lesions were taken 72 hpi. (B) Relative pathogen biomass in inoculated etiolated hypocotyls expressed as the ratio between the amounts of *P*. *sojae* DNA and soybean DNA detected at 72 hpi with the ratio P6497/soybean set at 1. (C) Relative numbers of sporangia with the number produced in wild-type strain P6497 set at 1. All experiments were repeated three times with similar results. Asterisks indicate significant differences at *P<0*.*01* (**), *P<0*.*05* (*). (D) Western blot analysis for all the strains mentioned above. Total proteins were extracted from these strains and SDS-PAGE and immunoblots were performed with GFP or FLAG antibodies.

To assess whether the altered localization pattern of PsYPK1 was related to its function, we generated *P*. *sojae* tranformants in which the N-terminal region of PsYPK1 is deleted and used one of the lines, named PsYPK1^ΔN^, in infection assays. This revealed that PsYPK1^ΔN^ has a decreased virulence on soybean (**Fig 6A, 6B and 6D**), albeit that this decrease is far less than that of the full-length knockout (**Fig 3)**. Notably, sporangium production in PsYPK1^ΔN^ was dramatically increased (**Fig 6A, 6C and 6D**) as opposed to the significant decrease in sporangia production in the full-length knockout (**Fig 2**). These results indicate that the catalytic domain of PsYPK1 is sufficient for sporangium formation. For further confirmation of the essential role of the kinase domain, we generated a mutant in which the kinase domain of PsYPK1 is deleted (PsYPK1^ΔC^). Similar to the full length PsYPK1-knockout (ΔPsYPK1), PsYPK1^ΔC^ produced less sporangia and less oospore formation, and showed reduced virulence (**S8A–S8C Fig**).

### PsGPA1 prevents nuclear localization of PsYPK1 and this leads to suppression of sporangia formation

Considering that the N-terminal region of PsYPK1 is required for interaction with PsGPA1, the nuclear localization of PsYPK1^ΔN^ might reflect the default steady-state distribution of PsYPK1. This led us to investigate to what extent PsGPA1 affects the localization of PsYPK1. To this end, we examined the localization of PsYPK1*-*GFP in *PsGPA1* overexpression and silenced strains. In the *PsGPA1* overexpression strain the fluorescence is more or less equally distributed with a slightly stronger signal close to the nucleus but not in the nucleus. Conversely, in the *PsGPA1*-silenced mutant the fluorescence in the nucleus is stronger than in the cytoplasm (**Fig 5A and 5B and S7D Fig**) suggesting that PsGPA1 prevents nuclear localization of PsYPK1.

This then raised the question whether the decreased level of PsYPK1 in the nucleus instigated by the presence of PsGPA1 is biologically relevant. After all, the PsYPK1 knock-out strain and PsGPA1 overexpressing strain have similar phenotypes, i.e. decreased sporangium formation and reduced virulence on *P*. *sojae* (**Figs 3 and 4**). To investigate this, we generated transformants co-expressing *PsGPA1* and *PsYPK1* and analyzed their phenotypes. To eliminate the influence of the native PsYPK1 in the wild-type, we used a PsYPK1 knockout strain as recipient strain and included a complemented PsYPK1 knockout strain as control. Consistent with the localization change described above, complementation with PsYPK1 was found to rescue the virulence defects and the sporangium production. In contrast, co-expression of *PsGPA1* and *PsYPK1* resulted in a significant decrease in sporangia numbers and reduced virulence on soybean (**Fig 6A–6D**). To further confirm that this phenotype is dependent on the interaction between PsGPA1 and PsYPK1, we also co-expressed *PsGPA1* and *PsYPK1*^*ΔN*^ in the PsYPK1 knockout strain. PsYPK1^ΔN^ can no longer interact with PsGPA1 but has retained its catalytic domain that is sufficient for sporangia formation. In the PsYPK1^*ΔN*^ complemented strain, *PsGPA1* overexpression did not affect sporangia production and did not change the virulence (**Fig 6A–6D**). Taken together, these findings point, at least in part, to the negative role of *PsGPA1* in sporangium formation and support the conclusion that PsGPA1 prevents localization of PsYPK1 in the nucleus by binding to its N-terminal portion, thus regulating sporangium formation.

### The PsYPK1 knockout and PsGPA1 overexpression strains are sensitive to rapamycin

In *S*. *cerevisiae*, YPK1 is directly phosphorylated by the target of rapamycin (TOR) complex-2 (TORC2) and this TOR signaling is required for many plasma membrane- and cell wall-associated events [30]. To determine whether PsGPA1 can affect the phosphorylation state of PsYPK1, we expressed PsYPK1 in wild-type, PsGPA1 overexpression or silenced strains. The phosphorylation state of PsYPK1 was tested by Pro-Q diamond phosphoprotein gel staining which can specifically reveals phosphorylated protein. In this study we included, the phosphorylated band of PsYPK1 show no changes in PsGPA1 overexpression or silenced strains compared with wild-type strain, while an obvious reduction was found in PsYPK1 incubating with phosphatase (**S8D Fig**), suggesting that PsGPA1 does not affect the phosphorylation state of PsYPK1.

To further insight to whether PsYPK1 is associated with TOR signaling in *P*. *sojae*, a rapamycin based growth assay was conducted. The PsGPA1 silenced mutant (PsGPA1-M27) showed the same growth rate as wild-type (P6497) with limited sensitivity to rapamycin, while a much higher growth inhibition was observed in both the ΔPsYPK1 and PsGPA1-OE strains (**S9 Fig**). In addition, growth assays of the same strains were performed upon exposure to oxidative, osmotic or cell wall stresses but none of these stresses had a significant effect (**S10 Fig**). Although these results point to involvement of PsYPK1 in TOR signaling, there is no evidence for requirement of PsYPK1 to cope with stress conditions.

### PsGPA1 and PsYPK1 co-regulate transcription of genes associated with sporangia formation in *P*. *sojae*

**Fig 7:**
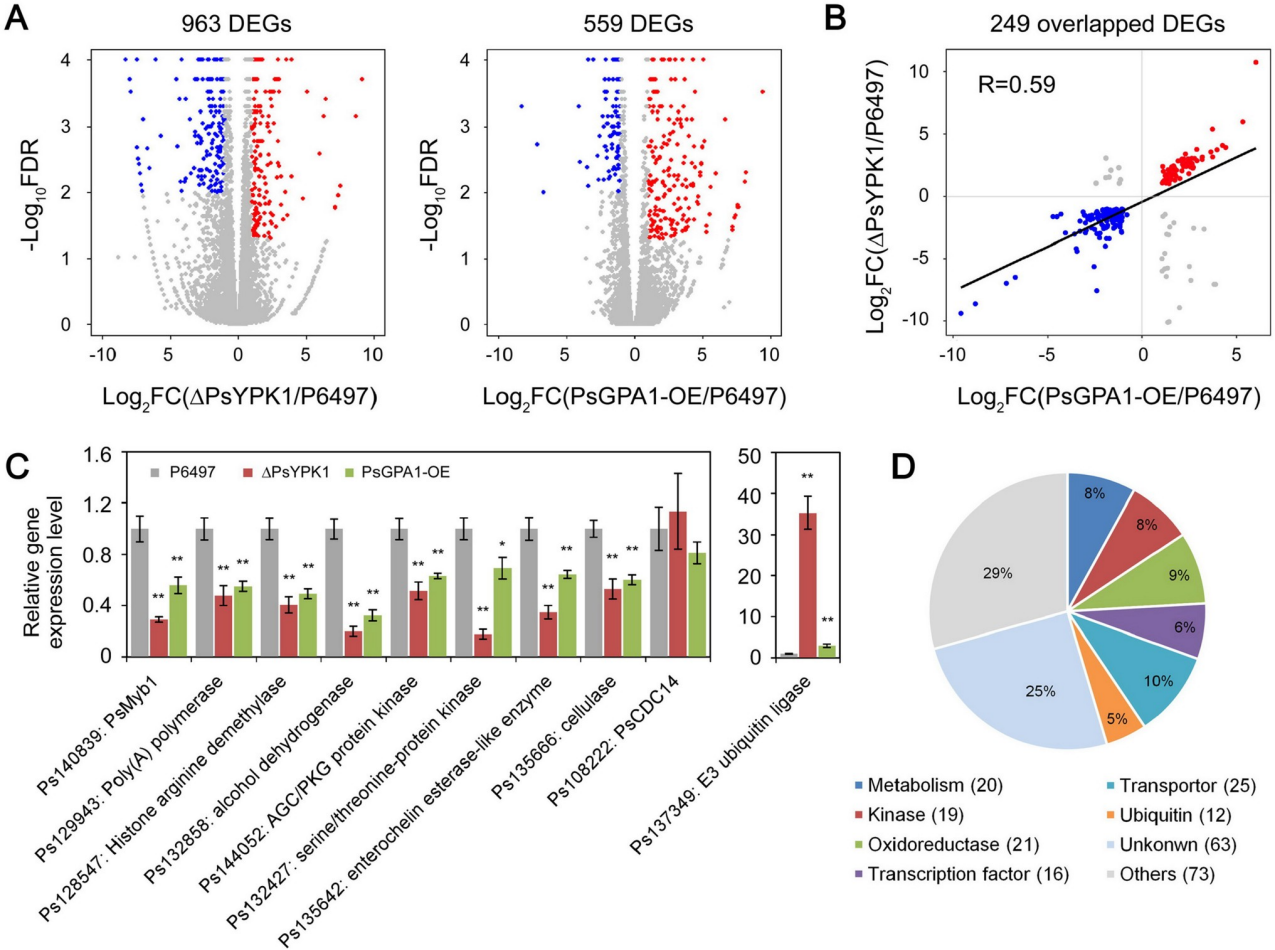
Overlap in genes differently expressed in the *PsGPA1* overexpression strain and *PsYPK1* knockout mutant. (A) Overview of differentially expressed genes (DEGs) in the *PsYPK1* knockout and *PsGPA1* overexpression strains, compared to wild-type (P6497) and (B) comparison of the consistency of DEGs between the *PsYPK1* knockout mutant and wild-type (ΔPsYPK1 vs. P6497) and the *PsGPA1* overexpression strain and wild-type (PsGPA1-OE vs. P6497). Red dots, upregulated genes; blue dots, downregulated genes. (C) Verification of expression levels of selected genes by qRT-PCR. Standard error bar (SD) represents relative expression level calculated by qPCR using the 2^-ΔΔCT^ method. The level of gene expression in the wild-type was set equal to 1 and used to calculate the relative expression levels of the genes in the transformants. All experiments were repeated three times with similar results. Asterisks indicate significant differences at *P<0*.*01* (**), *P<0*.*05* (*). (D) Functional annotation and classification of co-differentially expressed genes in *PsGPA1* overexpression and *PsYPK1* knockout strains.

It has been demonstrated in *S*. *cerevisiae*, that the protein kinase Ypk1 plays an indirect role in transcriptional regulation [31, 32]. Considering that PsYPK1 directly interacts with PsGPA1 and that they both affect sporangia formation, we examined whether PsGPA1 and PsYPK1 are engaged in regulating genes whose expression is associated with sporangia formation in *P*. *sojae*. We utilized RNA-Seq to analyze the genome-wide expression during sporangia formation in the *PsYPK1* knockout mutant, the *PsGPA1* overexpression strain, and the wild-type P6497 strain. Two independent biological replicates were analyzed by RNA-Seq and the strong correlation of gene expression levels between the replicates indicates that the data are reliable (**S11A Fig**). Of the more than 27 million transcript reads approximately 80% could be mapped onto the *P*. *sojae* genome. Compared to the wild-type, 963 and 559 differentially expressed genes (DEGs) were identified in ΔPsYPK1 and PsGPA1-OE, respectively (**Fig 7A and S3 Table**) with 249 DEGs overlapping between the two strains. The majority of these overlapping DEGs exhibited a good concordance with a correlation coefficient (R) of 0.59 (**Fig 7B**) and it is conceivable that these DEGs have a role in sporangium formation.

To further validate the reliability of the RNA-Seq data, 10 genes were selected for qRT-PCR analyses. This included genes whose expression in ΔPsYPK1 and PsGPA1-OE was either upregulated (Ps137349), downregulated (Ps140839, Ps129943, Ps128547, Ps132858, Ps144052, Ps132427, Ps135642, Ps135666), or unchanged (Ps108222) in comparison to wild-type. For all 10 genes the expression profiles obtained by qRT-PCR showed similar patterns of up- and downregulation or no changes (**Fig 7C**), thus supporting the reliability of the RNA-Seq data.

By GO annotation the 249 overlapping DEGs genes were, amongst others, classified as transcription factors (6%), metabolism-related proteins (8%), transporters (10%), kinase proteins (8%), and oxidoreductase-related proteins (8%) and E3 ubiquitin-protein ligases (5%) (**Fig 7D**). Most of these were significantly downregulated with the exception of the E3 ubiquitin-protein ligases that mainly showed a drastically elevated expression (**Fig 7D and S3 Table**). To ascertain significant pathways among these DEGs, KEGG analyses were performed. When compared with the entire genome background two pathways (glycolysis and gluconeogenesis pathways, and starch and sucrose metabolism pathways) were found to be enriched in overlapping DEGs (**S12A Fig**), suggesting these primary pathways might have a role in sporangium formation.

To determine whether these DEGs function in sporangia generation, we focused on those known to participate in asexual reproduction in *Phytophthora* species. Notably, PsMYB1, a Myb transcription factor, was clearly downregulated in both the RNA-seq and qRT-PCR analyses (**Fig 7C**), and this is consistent with previous findings showing that PsMYB1 plays an essential role in sporangial development in *P*. *sojae* [33]. Also in *P*. *infestans* Myb transcription factors have been implicated in sporulation. Xiang & Judelson (2014) reported induction of Myb gene expression during sporulation and found that silencing of Myb2R3 results in suppression of sporulation [34]. A previous study revealed that *PsCDC14*, a gene encoding cdc14 phosphatase, a cell cycle regulator that regulates nuclear behavior at an early stage of sporulation and is required for sporulation in *P*. *infestans* and *P*. *sojae* [35, 36]. However, *PsCDC14* expression was also not impacted by deleting *PsYPK1* or overexpressing *PsGPA1* (**Fig 7C**). These gene expression profiles point to an unconventional G protein signaling route in which GPA1 acts in association with PsYPK1 to mediate the regulation of a Myb transcription factor. The results demonstrate that PsGPA1 and PsYPK1 are both active in the pathway regulating the expression of genes predicted to be required for sporangium formation.

## Discussion

Although G-protein signaling is ubiquitous in eukaryotes it is not uniform among organisms. Several basic components of G-protein signaling are highly conserved and this includes the Gα subunit of the heterotrimeric G-protein. Yet, there is a large variation in the number of different Gα subunits per species and also their downstream effectors. As a consequence, the pathways regulated by each of these individual Gα subunits vary. *Phytophthora* species are exceptional in this regard. They possess only a single Gα protein, and this by itself plays a central role in the life cycle and in virulence of these pathogens [26, 27]. Earlier studies have demonstrated that the Gα protein in *Phytophthora* controls zoospore motility and chemotaxis [26, 27] and that the encoding gene has a relatively high expression level in sporulating hyphae [27]. In the present study, we discovered the suppressive role of the Gα protein during the sporangium stage in *P*. *sojae*.

In both fungi and plants, the Gα protein can act as a negative regulator in several processes. In budding yeast Gα protein Gpa1 directly binds to the mating-specific MAPK Fus3 to downregulate the mating signal [37, 38]. *Aspergillus fumigatus* has two Gα subunits GpaA and GpaB, that transduce signals leading to inhibition or stimulation of conidiation, respectively, while activating vegetative growth through modulation of the activity of a protein kinase A, i.e. PKAC1 [39, 40]. *Arabidopsis* GPA1 interacts with phospholipase Da1 (PLDa1) and inhibits its activity by coupling with guanosine diphosphate [41], and soybean Gα proteins are negative regulators of nodule formation [42]. The inherent nature of the Gα protein includes multiple modes of regulation in which Gα can transduce the signal to negatively or positively regulate downstream pathways thus allowing for an extremely high degree of plasticity in signal responses mediated by G proteins. In our current study we show that the Gα protein in *P*. *sojae* acts as a negative regulator of sporangium development but to accomplish this, it exploits the serine/threonine kinase PsYPK1. We also show that this kinase physically interacts with PsGPA1 and thus can be considered as an immediate downstream effector of PsGPA1. Furthermore, we found that PsYPK1 contributed to sporangium formation, whereas PsGPA1 suppresses it.

PsYPK1 is a member of the AGC (PKA-, PKG- and PKC- related) family of serine/threonine kinases and homologous to *S*. *cerevisiae* YPK1. ScYPK1 and ScYPK2 are functionally redundant paralogs that represent a distinct class of fungal AGC kinases [43, 44]. They both have an extended N-terminal region that has no detectable similarity to any characterized sequence motif or structural domain. Similarly, we found that PsYPK1 and its orthologs in other *Phytophthora* spp. have an extended N-terminal region that precedes the conserved C-terminal catalytic domain. Compared to YPK orthologs in other organisms the *Phytophthora* YPKs have the longest N-terminal region (**S3 Fig**). Despite the lack of a known domain the N-terminal region plays an important role. When deleted the localization of PsYPK1 changes with a higher abundance in the nucleus and the number of sporangia increases pointing to a negative role of the N-terminal region during in sporangium formation (**Figs 5 and 6**).

Such a regulatory effect has also been demonstrated for the N-terminal region of YPK1 in *S*. *cerevisiae*. For example, overexpression of a truncated version of YPK1 lacking the entire N-terminal region YPK1 (YPK1^ΔN^) was found to be toxic, whereas overexpression of the full-length *YPK1* or a kinase-dead *YPK1*^*ΔN*^ was not toxic. Moreover, consistent with our findings on PsYPK1, Roelants et al. showed that the N-terminal region of YPK1 is a determinant of its subcellular localization [45]. They found that the full-length YPK1 is exclusively localized to the cytosol, whereas the catalytic domain of YPK1 (YPK1^ΔN^) localizes predominantly to the nucleus [45]. The relevance of the N-terminal region is further supported by the observation that a mutated form of *YPK1* with a single substitution mutation in the N-terminal region was able to rescue the nonviability of a tor2^ts^ mutant, suggesting that the alteration of the N-terminal domain alleviates the need for TORC2-dependent phosphorylation of YPK1 [46]. As yet, however, regulators of YPK1/2 that act via direct interaction with the N-terminal region have not been identified. Our study in *P*. *sojae* is the first to identify a protein, namely a Gα subunit, which interacts with the N-terminal region of YPK1 ortholog and that act as direct regulator of this YPK1.

We demonstrated that PsGPA1 physically interact with PsYPK1 and that both are involved in sporangium formation. Knockout mutants (ΔPsYPK1) showed strongly reduced sporangia production, a phenotype that could be rescued by complementation with PsYPK1. In contrast, targeted deletion of the N-terminal region of PsYPK1 (PsYPK1^*ΔN*^) or *PsGPA1* overexpression resulted in increased sporangia production. The observation that *PsGPA1* overexpression abolished complementation of ΔPsYPK1 by PsYPK1 but not by PsYPK1^*ΔN*^ (**Fig 6**) is a strong indication that PsYPK1 regulates sporangium formation in *P*. *sojae* through PsGPA1-dependent and independent mechanisms. To verify this hypothesis we compared the genome wide expression during sporangia formation in the ΔPsYPK1 strain and the *PsGPA1* overexpressing strain and found that indeed, there is a large overlap in genes that are up- or downregulated in both strains when compared to the wild-type strain. The noteworthy downregulated genes seem to be involved in a range of functions and included genes or gene families that were previously identified as being associated with sporulation. One example is a Myb transcription factor that was shown to be required for sporangial development in *P*. *sojae* [33]. Our data suggest that this Myb transcription factor functions downstream of the kinase PsYPK1. Also many dehydrogenases appeared to be downregulated based on the RNA-Seq analysis and this is in line with the report of Kim & Judelson who found an higher expression of dehydrogenase encoding genes in sporangia than in nonsporulating hyphae [47]. Moreover, the dramatically decreased expression of carbohydrate-active enzymes, including glycosyl hydrolase, cellulose, glucosidase, and carbohydrate-binding protein in both the ΔPsYPK1 and PsGPA1 overexpressing strains, is consistent with the finding that TORC2-Ypk1 action was connected to NADPH requirement and the pentose phosphate pathway [48].

Apart from the overlap in up- and downregulated we found many genes that are differentially expressed between the PsGPA1 overexpressing strain and ΔPsYPK1 mutant. In addition, comparing to overlapping DEGs, a larger number of DEGs from ΔPsYPK1 mutant enriched in metabolic pathways (**S12B Fig**). This is not unexpected as it aligns with the idea that PsYPK1 functions in both PsGPA1-dependent and independent pathways and with the incomplete matching of phenotypes of the two strains (**Fig 7D and S4B Fig**). For example, in the ΔPsYPK1 strain, many more carbohydrate-active enzymes were found to be downregulated, which may explain the increased severity of defects observed in this strain. Furthermore, from the virulence phenotypes it is also obvious that PsGPA1 and PsYPK1 affect the virulence of the pathogen at different levels. For PsGPA1 mutants, the reduction in virulence is mainly caused by a defect in chemotaxis and zoospores are no longer specifically attracted by the host plant soybean [27, 28]. It thus seems that PsGPA1 has a major role in an early infection stage with zoospores as infectious propagules (**S6C and S6D Fig**). The reduction of virulence in PsYPK1 mutants is mainly due to defects in the growth of invasive hyphae (**Fig 3C and 3D**). Therefore, the PsYPK1 mutant exhibits reduced virulence under all infection conditions (**S6C and S6D Fig**).

In conclusion, we propose a new model addressing the role of the G protein α subunit in the asexual development of *P*. *sojae*. In this model Gα acts as a negative regulator by binding to the N-terminal region of PsYPK1, an AGC type serine/threonine kinase that is essential for sporangium production. This binding prevents localization of PsYPK1 in the nucleus, thereby repressing expression of its target genes which are likely essential for sporangium formation.

## Materials and methods

### *P*. *sojae* strains and culture conditions

The genome-sequenced *P*. *sojae* strain P6497 (Race 2), generously provided by Dr. Brett Tyler (Department of Botany and Plant Pathology, Oregon State University, Corvallis, OR, USA), served as the wild-type strain, and all transgenic lines in this study were routinely grown on 10% V8 agar medium at 25°C in the dark (Erwin and Ribeiro, 1996). The asexual life stages, such as vegetative hyphae, sporulating hyphae, zoospores, cysts, and germinated cysts, were collected as previously described [27]. The growth rates of the transformants were calculated using cultures growing on Plich agar medium. Plich medium is minimal medium with poor nutritional content. It contains per liter 0.5 g KH_2_PO_4_, 0.25 g MgSO_4_∙7H_2_O, 1 g asparagine, 1 mg thiamine, 0.5 g yeast extract, 10 mg b-sitosterol, 25 g glucose, and 15 g agar (26, 48, 49). Sporulating hyphae were prepared by repeatedly washing 2-day-old mycelia grown in 10% (v/v) V8 broth with sterile distilled water (SDW), followed by incubation in the dark at 25°C for 4–8 h, until sporangia developed. Zoospores were produced as described above.

### Co-immunoprecipitation (Co-IP) for selecting PsGPA1 interacting proteins

Constructs carrying 3×FLAG-tagged PsGPA1 and 3×FLAG-tag (control) in the vector pTOR were transformed into *P*. *sojae*. Stable *P*. *sojae* transformants were selected for total protein isolation and total protein lysates were analysed by western blotting using anti-FLAG antibodies (Abmart Inc., Shanghai, China). This revealed a band of 42 kDa, representing the PsGPA1-3×FLAG fusion protein that was present in the transformants, but not in the control (**S1A Fig**). Protein lysate from sporulating hyphae of PsGPA1-3×FLAG expressing transformants was mixed with anti-FLAG M2 beads. The bead-bound proteins were then eluted and analyzed by MS.

### Targeted gene deletion and complementation

Gene deletion mutants were generated using the CRISPR-mediated gene replacement strategy [6]. The *NPTII* gene ligated with two 1.0-kb fragments flanking the target gene was used as donor DNA in homology-directed repair (HDR) (**S4B Fig**). We transformed *P*. *sojae* using PEG-mediated protoplast transformation [27]. Putative transformants were selected by growth on 10% (v/v) V8 medium containing 50 μg/mL geneticin and screened by PCR using primer combinations as shown in S2B Fig. Primer set F1/R1 (**S2 Table**) located within the *PsYPK1* ORF was used to screen for deletion of PsYPK1 in the genome of resistant transformants. Primer sets F2/R2 and F3/R3 (**S2 Table**) were used to detect homologous recombination events (**S4B and S4C Fig**). HDR events were analysed by Sanger sequencing to confirm that the *PsYPK1* ORF was cleanly replaced (**S4D Fig**) and qRT-PCR was used to demonstrate absence of PsYPK1 (**S4B and S4E Fig**).

For complementation, the knockout mutant was transformed using *NPTII* as a selection marker and with a construct containing the entire gene coding region with mutated sgRNA sites inserted into the pTOR vector.

### RNA extraction and gene expression analysis

Total RNAs from distinct stages of the life cycle of *P*. *sojae* (vegetative hyphae, sporulating hyphae, zoospores, cysts, germinated cysts and stages of infection) were extracted as described previously [27, 49]. RNA integrity was tested via agarose gel electrophoresis. Prior to cDNA synthesis, all RNA samples were DNase I-treated using a DNase kit, following the manufacturer’s protocol. First strand cDNA was synthesized from 1–5 μg of total RNA using oligo (dT) primers with an M-MLV reverse transcriptase kit (Takara Bio, Inc.), following the manufacturer’s protocol. Semi-RT-PCR was performed to amplify the actin A gene (ACT-RTF/R) and *PsYPK1* (PsYPK1-QRT-F/R). SYBR green qRT-PCR assays were used to measure *PsYPK1* expression with the aid of the primer pair PsYPK1-QRT-F/R (**S2 Table**). All reactions were performed on an ABI 7500 Fast Real-Time PCR System (Applied Biosystems, Inc., Foster City, CA, USA). The results were analyzed using ABI 7500 sequence detection software. Relative gene expression levels were determined using actin A (primers ACT-RTF/R S2 Table) levels as internal controls. Means and standard deviations were calculated using data from three replicates.

RNA for RNA-seq analyses was isolated from mycelium that was cultured on 10% V8 broth at 25°C in the dark for 2 days. The mycelium was rinsed twice with sterile distilled water and flooded with sterile distilled water. After 6 h, mycelium was collected for RNA extraction.

### Growth, oospore and sporangium production

To determine growth rate, all strains were grown on V8 and Plich medium at 25°C in the dark. Colony diameters on Plich medium were measured during 7 days and on V8 medium during 4 days and the average growth rates were calculated (mm/day).

To monitor and quantify oospore production, strains were grown on lima bean agar (LBA) medium at 25°C in the dark. After 10 days the cultures were examined by microscopy. Three blocks of 1 cm^2^ were cut from the medium and in each block three random fields at 40×magnification were selected for counting the number of oospores.

To quantify sporangia production 10% V8 broth was inoculated with three round mycelial disks (7 mm in diameter) cut from a culture, and incubated for 48 h at 25°C in the dark. The mycelia were then rinsed twice with sterile distilled water, then flooded with sterile distilled water and left for 8 h to stimulate sporangia formation. This was then gently mixed in a blender to obtain a homogenous mixture. Subsequently, three random samples of 100 μl were taken and by microscopic examination at 40×magnification the number of sporangia in each sample was counted. All assays were repeated at least 3 times.

### Virulence assays

The soybean cultivar Hefeng 47, which is susceptible to *P*. *sojae* strain P6497, was grown in plastic pots containing vermiculite at 25°C for 4 days in the dark. Zoospores were retrieved as described previously [27] and diluted to a concentration of 100 zoospores/10 μl. Etiolated seedlings were inoculated by pipetting 10 μl of the zoospore suspension on the hypocotyls, and maintained in a climate-controlled room at 25°C and 80% relative humidity in the dark.

Manifestations of pathogenicity were evaluated at 3 dpi, and photographs were taken. To allow microscopic observation of the penetration of, and infectious hyphal expansion within soybean tissue, infected epidermal cells were collected at 12 and 24 hpi and soaked in Trypan Blue. After destaining in chloral hydrate and water, the infected epidermis cells were examined under a light microscope. Each strain was tested using at least two different preparations of zoospores, and five plants. All assays were repeated at least three times.

### Interaction assay *in vivo* and *in vitro*

For the *in vivo* assays, FLAG-tagged PsGPA1 and GFP-tagged PsYPK1 were stably co-expressed in *P*. *sojae*. Mycelia of co-expressing transformants were harvested and suspended in lysis buffer. The samples were centrifuged at 4°C for 10 min at 12,000 × *g* to remove debris and the protein lysates (supernatants) were transferred to a new tube. These were incubated at 4°C for 3–4 h with 25 μl of GFP-Trap_A beads (Chromotek, Planegg-Martinsried, Germany). The beads were then collected by centrifugation at 2500 × *g* and washed three times in 1 mL of washing buffer (per manufacturer’s recommendations). Bound proteins were boiled for 5 min and the presence of FLAG proteins was detected by western blotting using anti-FLAG M2-Peroxidase (HRP) antibody (#A8592; Sigma-Aldrich).

For the *in vitro* assays, the coding sequences of *PsYPK1* and *PsGPA1* were inserted into a pET28a vector (containing the His tag) and pGEX-4T-2 vector (containing the GST tag), respectively (GE Healthcare Life Science). The plasmids (pET28a empty vector, His-PsYPK1, GST empty vector, and GST-PsGPA1), were transformed to *E*. *coli* strain BL21 (DE3), protein expression was induced with 0.1 mM IPTG and after 12 hours of growth protein lysates were obtained. The pull-down assay was performed using the ProFound pull-down GST protein–protein interaction kit (Pierce) according to the manufacturer’s instructions. The soluble total GST-fusion proteins and His proteins were incubated with 25 μL glutathione agarose beads (Invitrogen) for 8 h at 4°C. Then, the beads were washed three times, and the presence of His proteins was detected by western blotting using anti-His antibody (Abmart, Inc.).

### Pro-Q analysis of protein phosphorylation

The Pro-Q Diamond Phosphoprotein Gel Stain (Invitrogen) selectively stains phosphoproteins in polyacrylamide gels. Total proteins were isolated from stable *P*. *sojae* transformants expressing PsYPK1-GFP-tagged proteins and incubated with anti-GFP affinity beads. Proteins bound to resins were eluted after a series of washing steps, as described in the manufacturer's instructions. The purified proteins were separated by SDS-PAGE and detected by a standard Pro-Q Diamond Phosphoprotein Gel Stain system (Invitrogen, P33300). The stained gels were visualized on a blue light trans-illuminator using a 532-nm laser and a 560-nm longpass as the excitation source and emission filter, respectively (Typhoon Trio^+^, Amersham Biosciences).

After obtaining results with Pro-Q Diamond Phosphoprotein Gel Stain, the total protein in the gel was stained with SYPRO Ruby Protein Gel Stain to ascertain the relative phosphorylation state of the proteins (Invitrogen, S12001).

### RNA-seq analysis

The resulting high-quality reads which had been filtered by fastQC were aligned to the complete genome assemblies of *Phytophthora sojae* (v1.1 for isolate P6497; jgi.doe.gov). A total of two mismatches and gaps per read were allowed, and only when both of a pair of reads were successfully mapped, the data was included in the analyses. Transcript abundance was indicated as Fragments Per Kilobase of exon model per Million mapped reads (FPKM). EdgeR software (www.bioconductor.org/packages/release/bioc/html/edgeR.html) was used to perform differential expression analysis between duplicate samples for all genes. To identify differentially expressed genes (DEGs), read counts for each gene model were obtained using featureCounts software (subread.sourceforge.net), then the log_2_ fold change (log_2_FC) value and adjusted *P* value were calculated using DESeq2 software (www.bioconductor.org/packages/release/bioc/html/DESeq2.html), and genes with an adjusted *P* value < 0.05 and an absolute |log_2_FC| ≥ 1 were considered differentially expressed.

## Supporting information

S1 Fig Co-immunoprecipitation of proteins interaction with PsGPA1. (A) Western blot analysis of lysates of *P*. *sojae* transformants carrying an empty vector (Flag) or a construct in which PsGPA1 is fused to a 3×Flag tag (PsGPA1-Flag) of the expression and immunoprecipitation using the FLAG antibody. The PsGPA1‐3×FLAG fusion protein detected by the Flag antibody is indicated by a red dot. (B) GST pull-down experiment showing that PsGPA1 physically interacts with Ps350990 (PsYPK1) and Ps350499 *in vitro*. GST-PsGPA1- or GST-bound resins were incubated with *E*. *coli* supernatant containing His-Ps350990 or His-Ps350499. The presence of His-tagged proteins was detected by western blot analysis using a His antibody.
(TIF)

S2 Fig Phylogenetic analysis of Ps350990 (PsYPK1) and its homologs in oomycetes. (A) Neighbour-joining tree of Ps350990 and its homologs from *Phytophthora sojae* (Red circle), *Phytophthora infestans* (Blue circle), *Phytophthora ramorum* (Fuchsia circle), *Hyaloperonospora parastica* (Green circle), *Pythium ultimum* (Cyan circle) and *Saprolegnia parastica* (Yellow circle), and ScYPK1/2 and its homologs in *S*. *cerevisiae* (Black rhombus). (B) Comparison of key sites for phosphorylation and kinase activity in ScYPK1, Ps350990 (PsYPK1) and Ps350990 homologs in *P*. *sojae*.
(TIF)

S3 Fig PsYPK1 has a long N-terminal region and a conserved C-terminal domain. (A) Phylogenetic tree based on the amino acid sequence of PsYPK1 (XP_009525581.1) and its orthologs from *Phytophthora infestans* (XP_002900048.1), *Phytophthora parasitica* (XP_008914324.1), *Saccharomyces cerevisiae* (NP_012796.1), *Homo sapiens* (NP_005618.2), *Fusarium graminearum* (XP_011324446.1), and *Arabidopsis thaliana* (OAP04608.1). On the left a schematic representation of the proteins in the seven species. The Ser/Thr kinase domain is represented by a gray box and the C-terminal region in AGC family kinases by a white box. (B) Sequence alignment of the kinase domains in PsYPK1 and its orthologs.
(TIF)

S4 Fig CRISPR-mediated gene replacement of *PsYPK1*. (A) Location of the primers used to screen the HDR mutants. (B) Analysis of genomic DNA from the wild-type (P6497), PsYPK1-knockout (ΔPsYPK1), empty vector control line (EV), complemented transformants (ΔPsYPK1-C1, C2), and empty control line of ΔPsYPK1 (ΔPsYPK1-EV) using the primers shown in (A) and actin primers as positive control. (C) Sanger sequencing traces of junction regions confirming that the *PsYPK1* ORF was precisely replaced. (D) Expression analyses of *PsYPK1* by reverse transcription-polymerase chain reaction (RT-PCR).
(TIF)

S5 Fig PsYPK1 is important for growth in nutrient poor medium. (A) Growth characteristics of the wild-type (P6497), PsYPK1-knockout (ΔPsYPK1), empty vector control line (EV), complemented (ΔPsYPK1-C1, C2) transformants, and empty control line of ΔPsYPK1 (ΔPsYPK1-EV) on Plich medium in which glucose is replaced with fructose, galactose, xylose or mannose. (B) Statistical analysis of the growth rate after 7 days. All experiments were repeated three times with similar results. Asterisks indicate significant differences at *P<0*.*01* (**).
(TIF)

S6 Fig Pathogenicity assays of the wild-type (P6497), *PsGPA1* silenced mutant (PsGPA1-M27), *PsGPA1* overexpression strain (PsGPA1-OE) and *PsYPK1*-knockout mutant (ΔPsYPK1). (A-B) RNA expression levels of *PsYPK1* (A) and *PsGPA1* (B) during the asexual life and stages of infection were measured by RNA sequencing (RNA-seq). Samples were collected from various life cycle stages including mycelia (MY), sporangium (SP), zoospores (ZO), cysts (CY), germinating cysts (GC) and ‘IF1.5 to IF24’materials (samples taken 1.5, 3, 6, 12 and 24 h after inoculating hyphae on soybean leaves). (C) Zoospores or hyphae of each strain were inoculated on soybean hypocotyls. In the panels labeled with ‘Zoospore wounded’ the hypocotyls were wounded prior to inoculation with zoospores. Photographs were taken at 3 days post inoculation (dpi) or 7 dpi. (D) Quantification of pathogen biomass. Relative biomass is expressed as the ratio between the amount of *P*. *sojae* DNA and soybean DNA. Asterisks indicate significant differences at *P<0*.*01* (**).
(TIF)

S7 Fig PsGPA1 and N-terminal region of PsYPK1 are both localized in the cytoplasm. (A) Microscopic analyses of *P*. *sojae* transformants expressing PsYPK1^N-ter^-GFP or PsGPA1-GFP in the wild-type recipient stain P6497 (marked as PsYPK1^N-ter^-GFP or PsGPA1-GFP, respectively). DAPI (4', 6-diamidino-2-phenylindole) staining was performed by adding DAPI to the cultures 5 min prior to the microscopic analysis. DIC: differential interference contrast; Merge: overlay of DIC, GFP fluorescence and DAPI staining. Bar, 20 μm. (B) Relative fluorescence intensity along the red arrows in A. Green line: PsYPK1^N-ter^-GFP or PsGPA1-GFP, blue line: nucleus (stained with DAPI). (C, D) Western blot analysis for fluorescence observation strains. Total proteins extracted from fluorescent strains (C: PsYPK1^N-ter^-GFP and PsGPA1-GFP; D: PsYPK1-GFP, PsGPA1-FLAG/PsYPK1-GFP, PsGPA1-M27/PsYPK1-GFP, PsYPK1^ΔN^-GFP) were subjected to SDS-PAGE and immunoblots were incubated with anti-GFP or anti-FLAG.
(TIF)

S8 Fig The kinase domain of PsYPK1 is important for sporangium and oospore production and virulence. (A) Comparisons of sporangium and oospore production, and pathogenicity tests on soybean hypocotyls in wild-type strain (P6497), empty control line (PsYPK1^ΔC^-EV), and the kinase domain deletion mutants (PsYPK1^ΔC^-M32, PsYPK1^ΔC^-M34, PsYPK1^ΔC^-M72). (B) Statistical analysis of all of the phenotypes discussed above. All experiments were repeated three times with similar results. Scale bar, 50 μm. Asterisk indicates significant difference at *P<0*.*01* (**). (C) Analysis of genomic DNA from all the strains mentioned above using the full-length primer of PsYPK1. (D) PsGPA1 cannot affect the phosphorylation state of PsYPK1. PsYPK1 protein was expressed in wild-type, PsGPA1 overexpression or silenced strains for the phosphorylation assay, or incubating with phosphatase as a control (marked as PsYPK1-GFP, PsGPA1-FLAG/PsYPK1-GFP and PsGPA1-M27/PsYPK1-GFP, PsYPK1-GFP/phosphatase, respectively) and the gel image was visualized after Pro-Q Diamond phosphoprotein gel staining. In all cases, the upper panel represents Pro-Q Diamond-stained phosphoprotein gel and the two bottom panels show the same protein samples using western blot analysis to display the PsYPK1-GFP or PsGPA1-FLAG protein. Numbers represent relative signal intensities. Western blot analysis of PsYPK1-GFP was used as a loading control.
(TIF)

S9 Fig *PsGPA1* overexpression and *PsYPK1* knockout strains are sensitive to rapamycin. (A) Growth characteristics of the wild-type (P6497), *PsGPA1* silenced mutant (PsGPA1-M27), *PsGPA1* overexpression strain (PsGPA1-OE) and *PsYPK1* knockout (ΔPsYPK1) on V8 agar medium with DMSO or 5, 20ng/ul rapamycin. (B) Statistical analysis of the growth rate after 4 days. All experiments were repeated three times with similar results. Error bars represent the standard deviation and asterisks denote significant differences (**P < 0.01; *P < 0.05).
(TIF)

S10 Fig The PsGPA1 and PsYPK1 mutants show no difference in oxidative, osmotic and cell wall stresses. (A) Growth characteristics of wild-type (P6497), *PsGPA1* silenced mutant (PsGPA1-M27), *PsGPA1* overexpression strain (PsGPA1-OE) and *PsYPK1* knockout mutant (ΔPsYPK1) on V8 agar medium only or supplemented with 2.5 or 5mM H_2_O_2_, 0.5 M sorbitol or 600ng/ul Congo red (CR). (B) Colony diameters were measured in each independent biological experiment after 4 days of growth. Rates of growth inhibition were calculated for each treatment relative to growth on V8 agar medium only.
(TIF)

S11 Fig Genome-wide expression analyses. (A) Global statistical assessment of biological replicates. Scattering matrix analysis of fragments per kilobase of exon model per million mapped reads (FPKM) from all of the transcriptome samples, including the wild-type (P6497), PsGPA1 overexpression (PsGPA1-OE1 and PsGPA1-OE2), and PsYPK1 knockout (ΔPsYPK-1 and ΔPsYPK1-2) samples. One of the P6497 samples failed in library construction, so the global statistical assessment data of the P6497 biological duplicate is absent. PsGPA1-OE1 and PsGPA1-OE2, and ΔPsYPK-1 and ΔPsYPK1-2 are biological duplicates. Variation analysis was conducted not only in independent biological replicates but also between different strains. (B, C) Functional annotation and classification of specific DEGs in the *PsYPK1* knockout (B) and *PsGPA1* overexpression (C) samples.
(TIF)

S12 Fig Statistics of pathway enrichment based on the Kyoto Encyclopedia of Genes and Genomes (KEGG) analysis. (A, B) The top 20 pathways enriched by overlapping DEGs (A) or DEGs from ΔPsYPK1 mutant (B) are displayed in an enriched scatter diagram. The rich factor, number of genes, and q value were used to measure the degree of KEGG enrichment. When a pathway with greater rich factor, larger number of genes and, q value is less than 0.05, the enrichment is more significant.
(TIF)

S1 Table Putative PsGPA1-interacting genes identified by co-immunoprecipitation and mass spectrometry analysis. (XLSX)

S2 Table Primers used in this study. (XLSX)

S3 Table Detailed information of differentially expressed genes. (XLSX)
