# Supplementary figures and images for: G protein α subunit suppresses sporangium formation through a serine/threonine protein kinase in Phytophthora sojae
Source: PLoS Pathog. 2020 Jan 21;16(1):e1008138. doi: 10.1371/journal.ppat.1008138 (PMC7010300; doi:10.1371/journal.ppat.1008138)

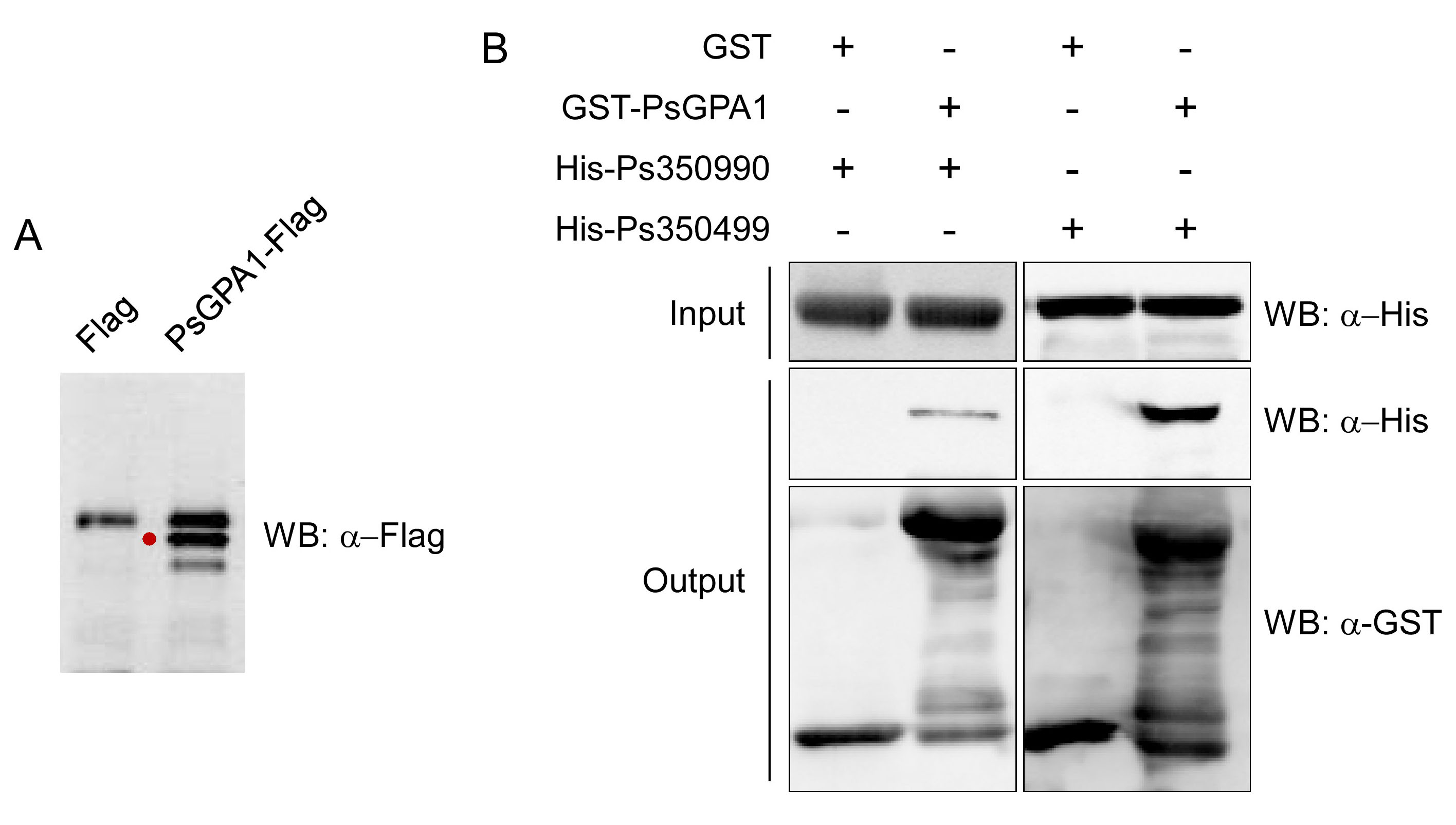

Supplement: S1 Fig — (A) Western blot analysis of lysates of P. sojae transformants carrying an empty vector (Flag) or a construct in which PsGPA1 is fused to a 3×Flag tag (PsGPA1-Flag) of the expression and immunoprecipitation using the FLAG antibody. The PsGPA1‐3×FLAG fusion protein detected by the Flag antibody is indicated by a red dot. (B) GST pull-down experiment showing that PsGPA1 physically interacts with Ps350990 (PsYPK1) and Ps350499 in vitro. GST-PsGPA1- or GST-bound resins were incubated with E. coli supernatant containing His-Ps350990 or His-Ps350499. The presence of His-tagged proteins was detected by western blot analysis using a His antibody. (TIF) [file ppat.1008138.s001.tif]

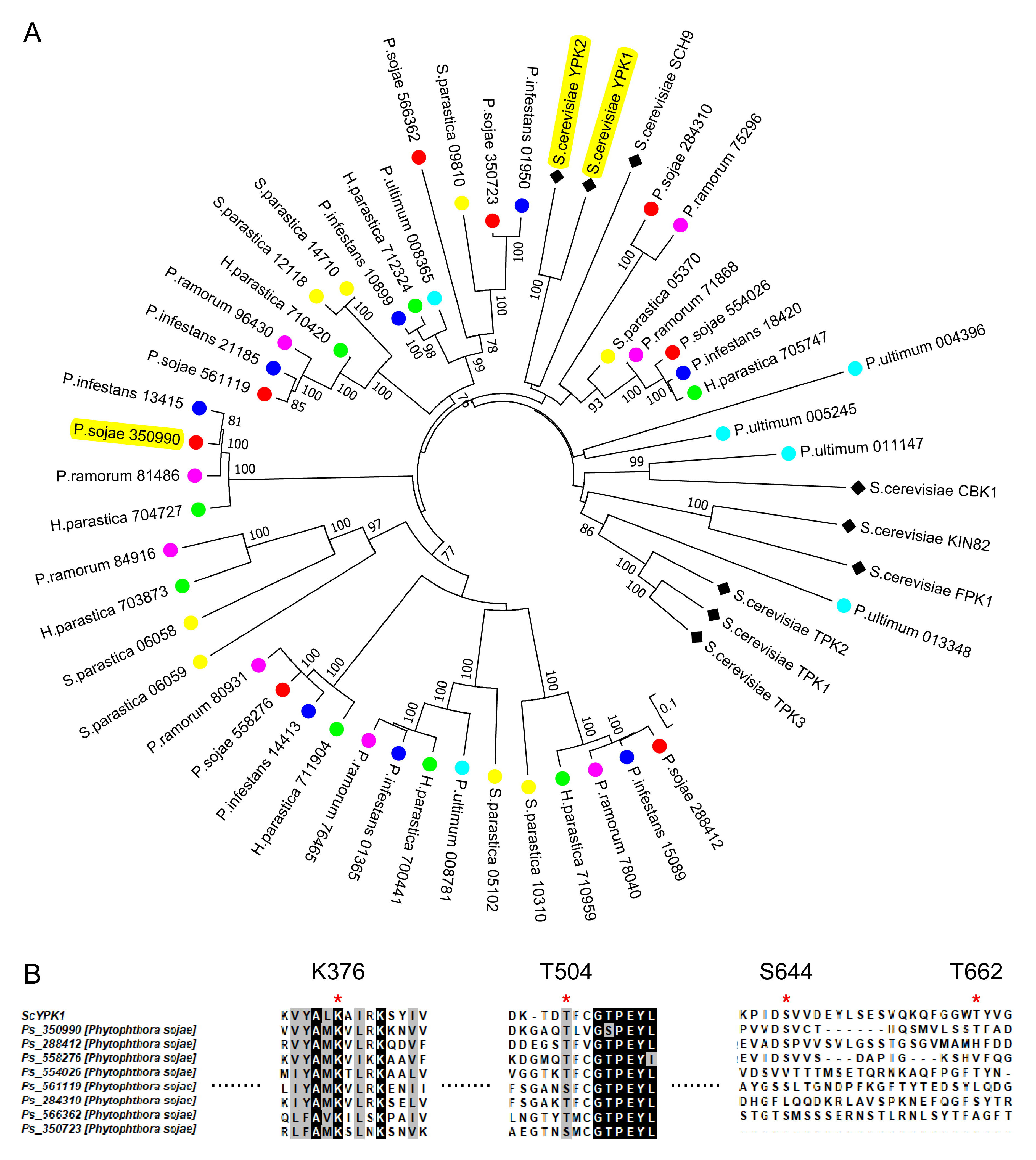

Supplement: S2 Fig — (A) Neighbour-joining tree of Ps350990 and its homologs from Phytophthora sojae (Red circle), Phytophthora infestans (Blue circle), Phytophthora ramorum (Fuchsia circle), Hyaloperonospora parastica (Green circle), Pythium ultimum (Cyan circle) and Saprolegnia parastica (Yellow circle), and ScYPK1/2 and its homologs in S. cerevisiae (Black rhombus). (B) Comparison of key sites for phosphorylation and kinase activity in ScYPK1, Ps350990 (PsYPK1) and Ps350990 homologs in P. sojae. (TIF) [file ppat.1008138.s002.tif]

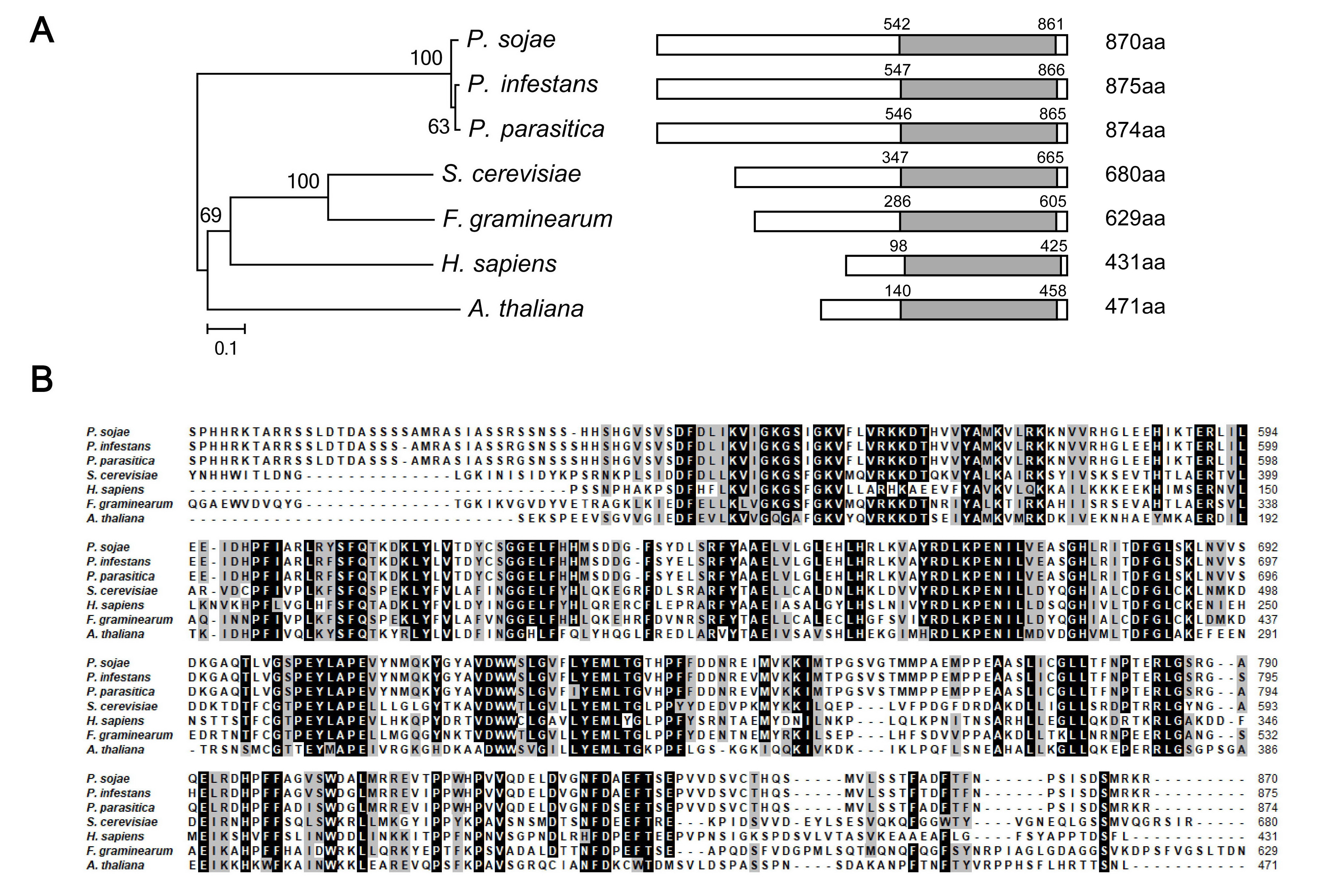

Supplement: S3 Fig — (A) Phylogenetic tree based on the amino acid sequence of PsYPK1 (XP_009525581.1) and its orthologs from Phytophthora infestans (XP_002900048.1), Phytophthora parasitica (XP_008914324.1), Saccharomyces cerevisiae (NP_012796.1), Homo sapiens (NP_005618.2), Fusarium graminearum (XP_011324446.1), and Arabidopsis thaliana (OAP04608.1). On the left a schematic representation of the proteins in the seven species. The Ser/Thr kinase domain is represented by a gray box and the C-terminal region in AGC family kinases by a white box. (B) Sequence alignment of the kinase domains in PsYPK1 and its orthologs. (TIF) [file ppat.1008138.s003.tif]

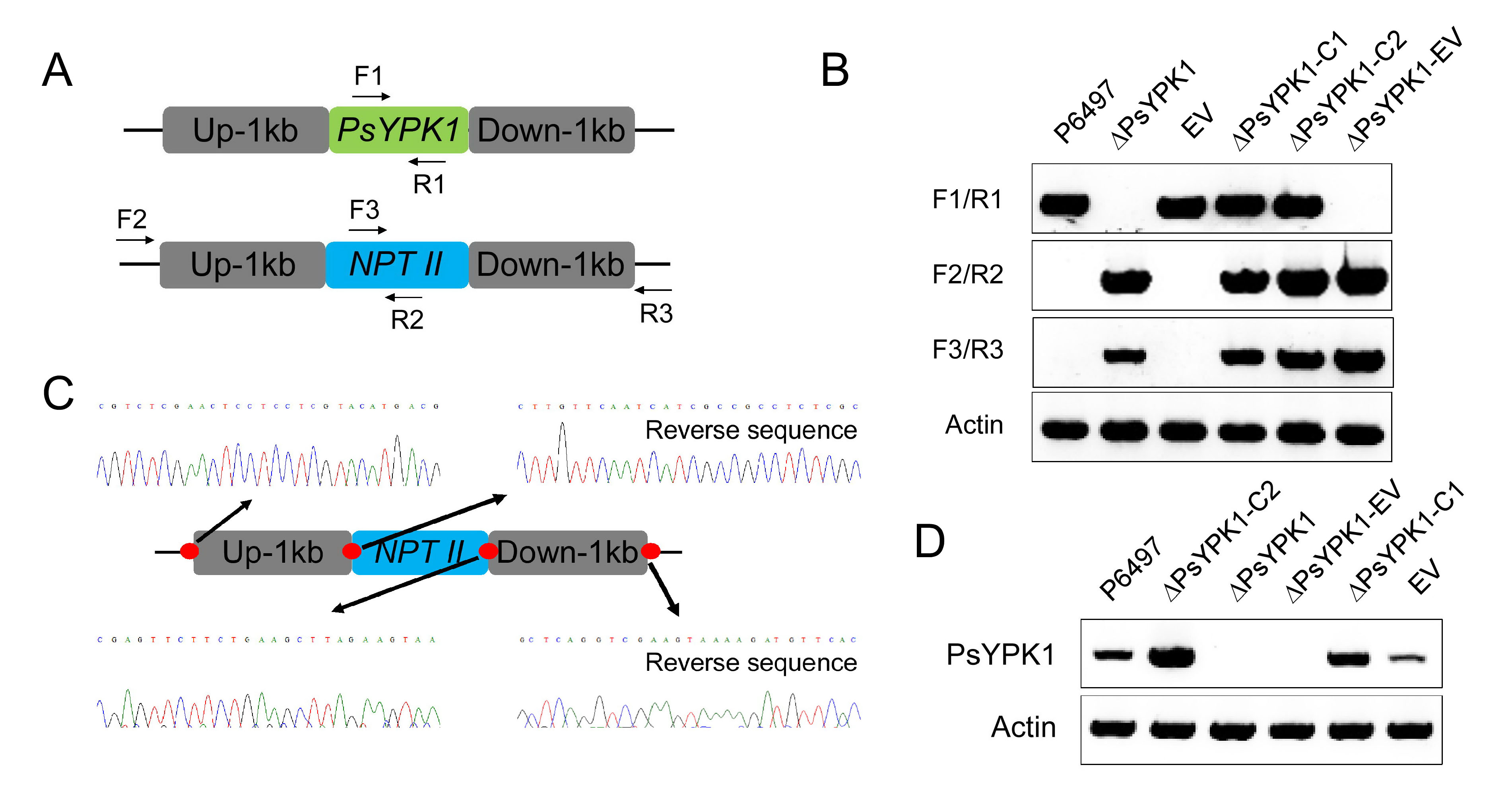

Supplement: S4 Fig — (A) Location of the primers used to screen the HDR mutants. (B) Analysis of genomic DNA from the wild-type (P6497), PsYPK1-knockout (ΔPsYPK1), empty vector control line (EV), complemented transformants (ΔPsYPK1-C1, C2), and empty control line of ΔPsYPK1 (ΔPsYPK1-EV) using the primers shown in (A) and actin primers as positive control. (C) Sanger sequencing traces of junction regions confirming that the PsYPK1 ORF was precisely replaced. (D) Expression analyses of PsYPK1 by reverse transcription-polymerase chain reaction (RT-PCR). (TIF) [file ppat.1008138.s004.tif]

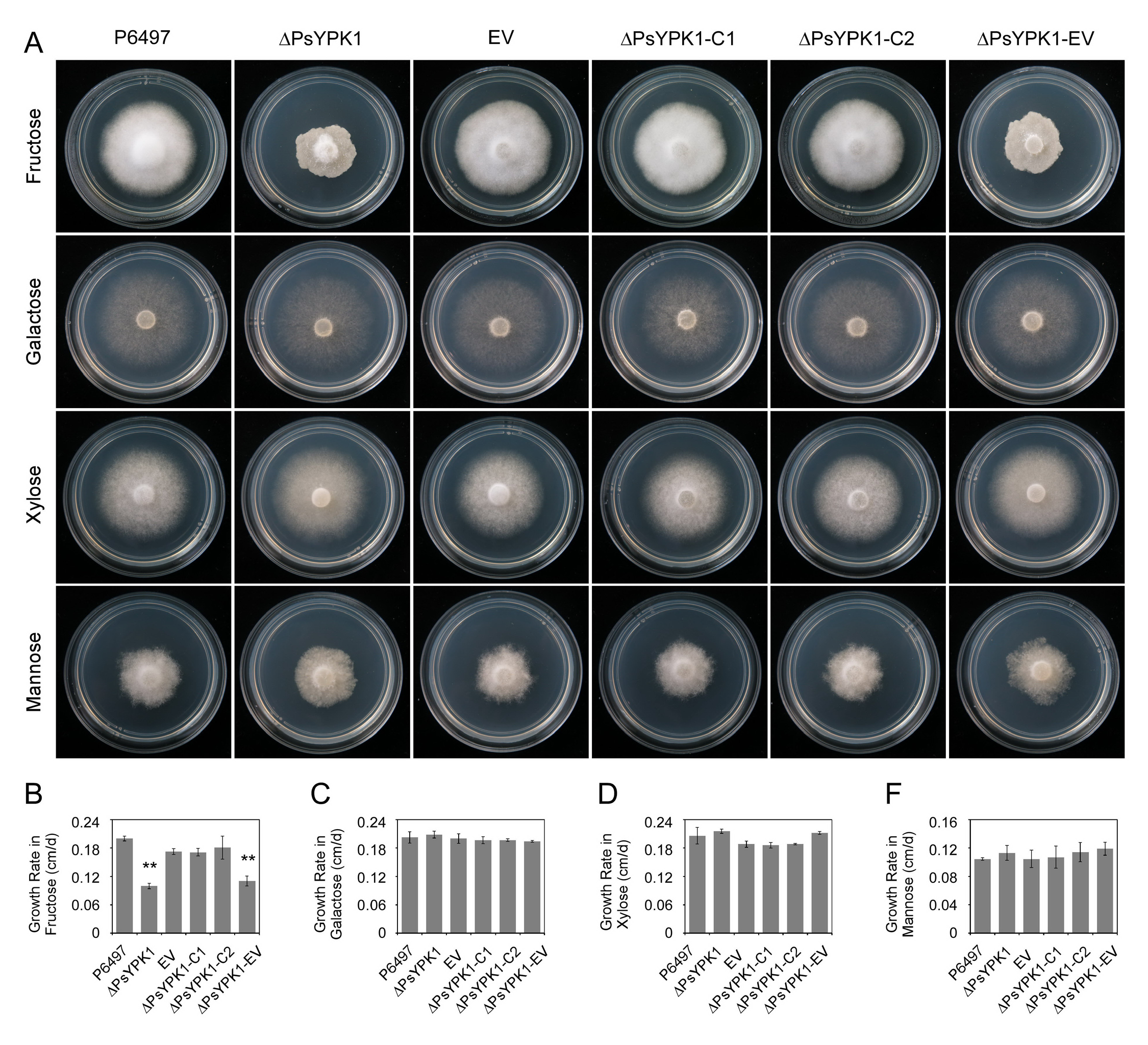

Supplement: S5 Fig — (A) Growth characteristics of the wild-type (P6497), PsYPK1-knockout (ΔPsYPK1), empty vector control line (EV), complemented (ΔPsYPK1-C1, C2) transformants, and empty control line of ΔPsYPK1 (ΔPsYPK1-EV) on Plich medium in which glucose is replaced with fructose, galactose, xylose or mannose. (B) Statistical analysis of the growth rate after 7 days. All experiments were repeated three times with similar results. Asterisks indicate significant differences at P<0.01 (**). (TIF) [file ppat.1008138.s005.tif]

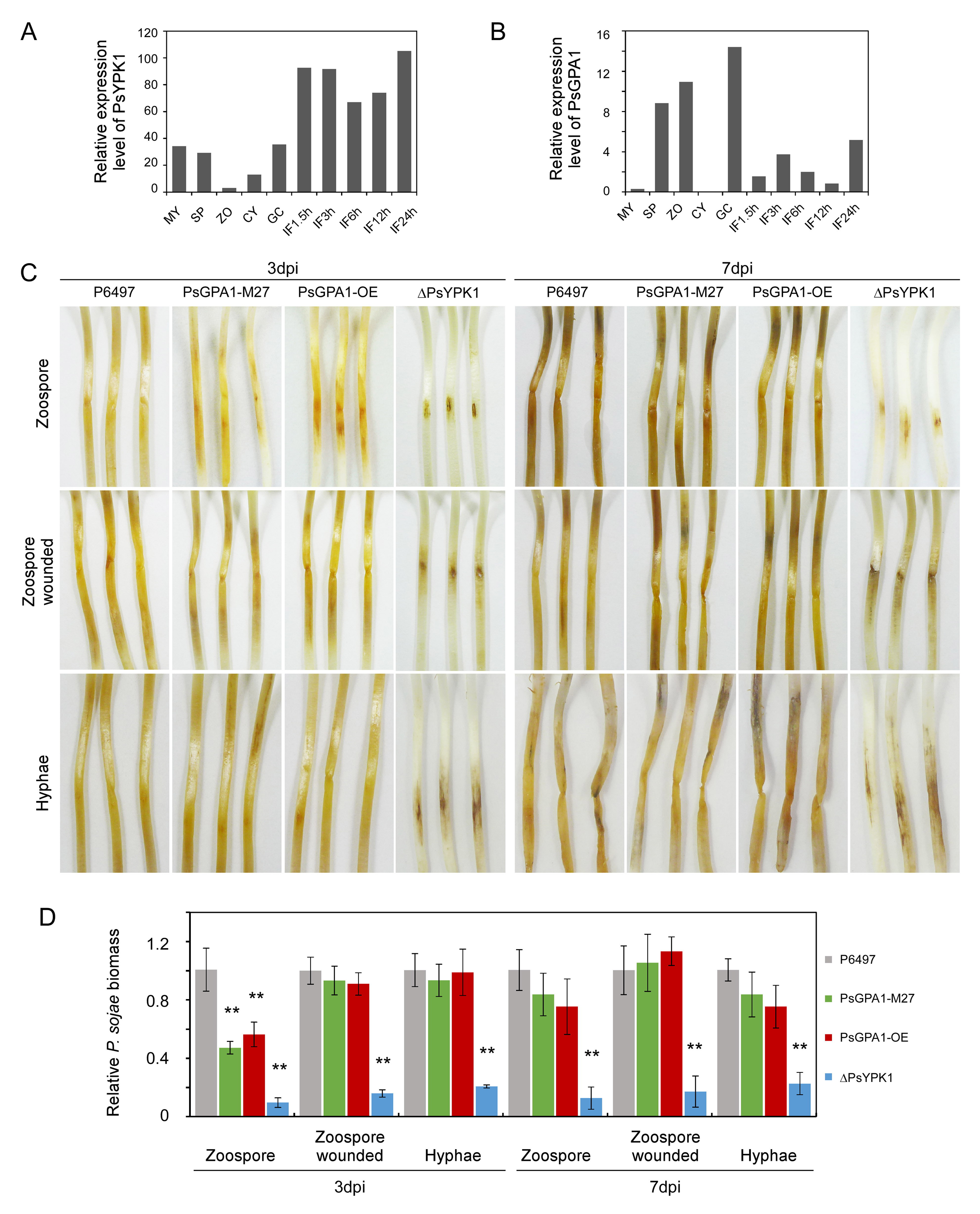

Supplement: S6 Fig — (A-B) RNA expression levels of PsYPK1 (A) and PsGPA1 (B) during the asexual life and stages of infection were measured by RNA sequencing (RNA-seq). Samples were collected from various life cycle stages including mycelia (MY), sporangium (SP), zoospores (ZO), cysts (CY), germinating cysts (GC) and ‘IF1.5 to IF24’materials (samples taken 1.5, 3, 6, 12 and 24 h after inoculating hyphae on soybean leaves). (C) Zoospores or hyphae of each strain were inoculated on soybean hypocotyls. In the panels labeled with ‘Zoospore wounded’ the hypocotyls were wounded prior to inoculation with zoospores. Photographs were taken at 3 days post inoculation (dpi) or 7 dpi. (D) Quantification of pathogen biomass. Relative biomass is expressed as the ratio between the amount of P. sojae DNA and soybean DNA. Asterisks indicate significant differences at P<0.01 (**). (TIF) [file ppat.1008138.s006.tif]

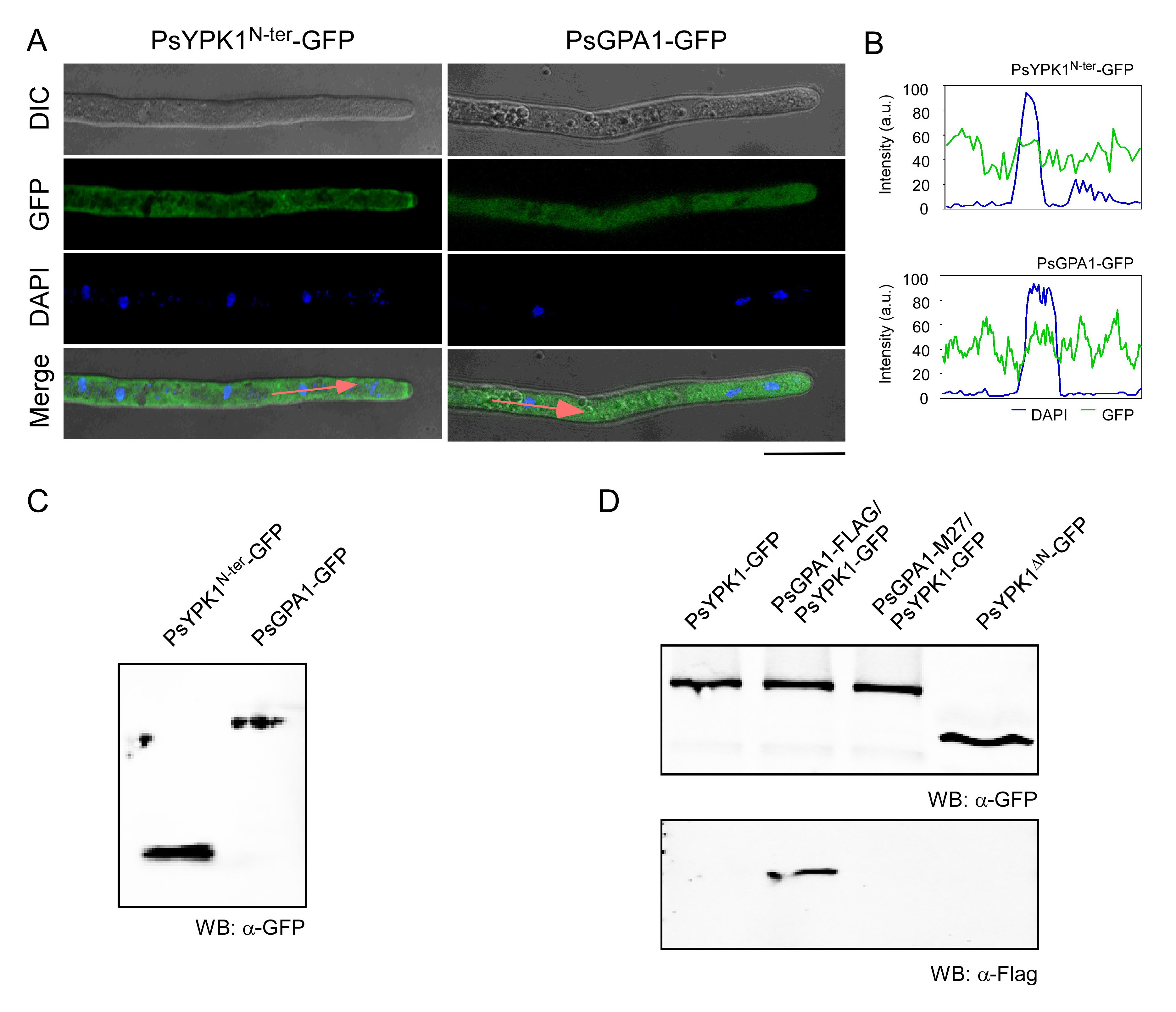

Supplement: S7 Fig — (A) Microscopic analyses of P. sojae transformants expressing PsYPK1N-ter-GFP or PsGPA1-GFP in the wild-type recipient stain P6497 (marked as PsYPK1N-ter-GFP or PsGPA1-GFP, respectively). DAPI (4', 6-diamidino-2-phenylindole) staining was performed by adding DAPI to the cultures 5 min prior to the microscopic analysis. DIC: differential interference contrast; Merge: overlay of DIC, GFP fluorescence and DAPI staining. Bar, 20 μm. (B) Relative fluorescence intensity along the red arrows in A. Green line: PsYPK1N-ter-GFP or PsGPA1-GFP, blue line: nucleus (stained with DAPI). (C, D) Western blot analysis for fluorescence observation strains. Total proteins extracted from fluorescent strains (C: PsYPK1N-ter-GFP and PsGPA1-GFP; D: PsYPK1-GFP, PsGPA1-FLAG/PsYPK1-GFP, PsGPA1-M27/PsYPK1-GFP, PsYPK1ΔN-GFP) were subjected to SDS-PAGE and immunoblots were incubated with anti-GFP or anti-FLAG. (TIF) [file ppat.1008138.s007.tif]

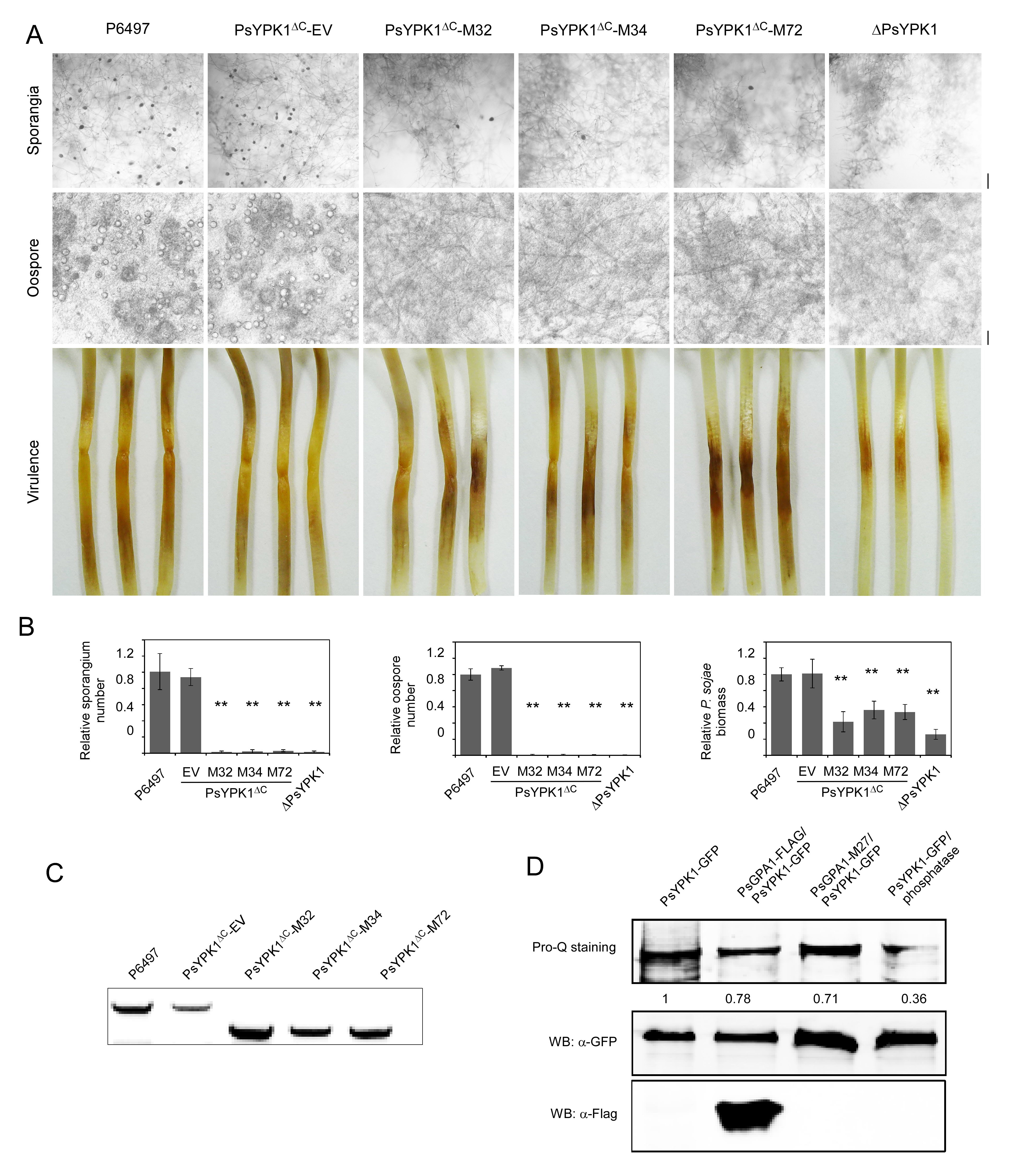

Supplement: S8 Fig — (A) Comparisons of sporangium and oospore production, and pathogenicity tests on soybean hypocotyls in wild-type strain (P6497), empty control line (PsYPK1ΔC-EV), and the kinase domain deletion mutants (PsYPK1ΔC-M32, PsYPK1ΔC-M34, PsYPK1ΔC-M72). (B) Statistical analysis of all of the phenotypes discussed above. All experiments were repeated three times with similar results. Scale bar, 50 μm. Asterisk indicates significant difference at P<0.01 (**). (C) Analysis of genomic DNA from all the strains mentioned above using the full-length primer of PsYPK1. (D) PsGPA1 cannot affect the phosphorylation state of PsYPK1. PsYPK1 protein was expressed in wild-type, PsGPA1 overexpression or silenced strains for the phosphorylation assay, or incubating with phosphatase as a control (marked as PsYPK1-GFP, PsGPA1-FLAG/PsYPK1-GFP and PsGPA1-M27/PsYPK1-GFP, PsYPK1-GFP/phosphatase, respectively) and the gel image was visualized after Pro-Q Diamond phosphoprotein gel staining. In all cases, the upper panel represents Pro-Q Diamond-stained phosphoprotein gel and the two bottom panels show the same protein samples using western blot analysis to display the PsYPK1-GFP or PsGPA1-FLAG protein. Numbers represent relative signal intensities. Western blot analysis of PsYPK1-GFP was used as a loading control. (TIF) [file ppat.1008138.s008.tif]

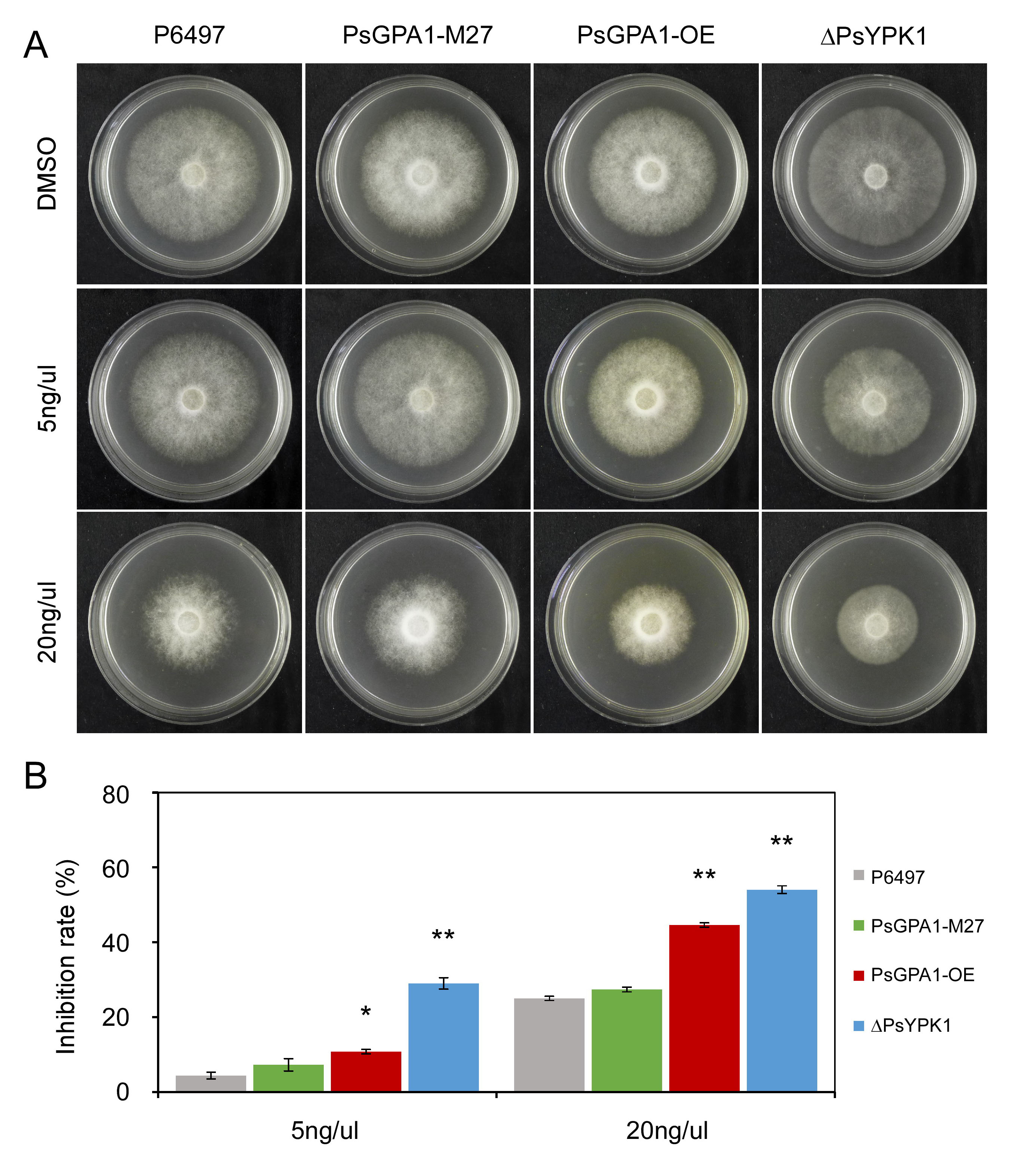

Supplement: S9 Fig — (A) Growth characteristics of the wild-type (P6497), PsGPA1 silenced mutant (PsGPA1-M27), PsGPA1 overexpression strain (PsGPA1-OE) and PsYPK1 knockout (ΔPsYPK1) on V8 agar medium with DMSO or 5, 20ng/ul rapamycin. (B) Statistical analysis of the growth rate after 4 days. All experiments were repeated three times with similar results. Error bars represent the standard deviation and asterisks denote significant differences (**P < 0.01; *P < 0.05). (TIF) [file ppat.1008138.s009.tif]

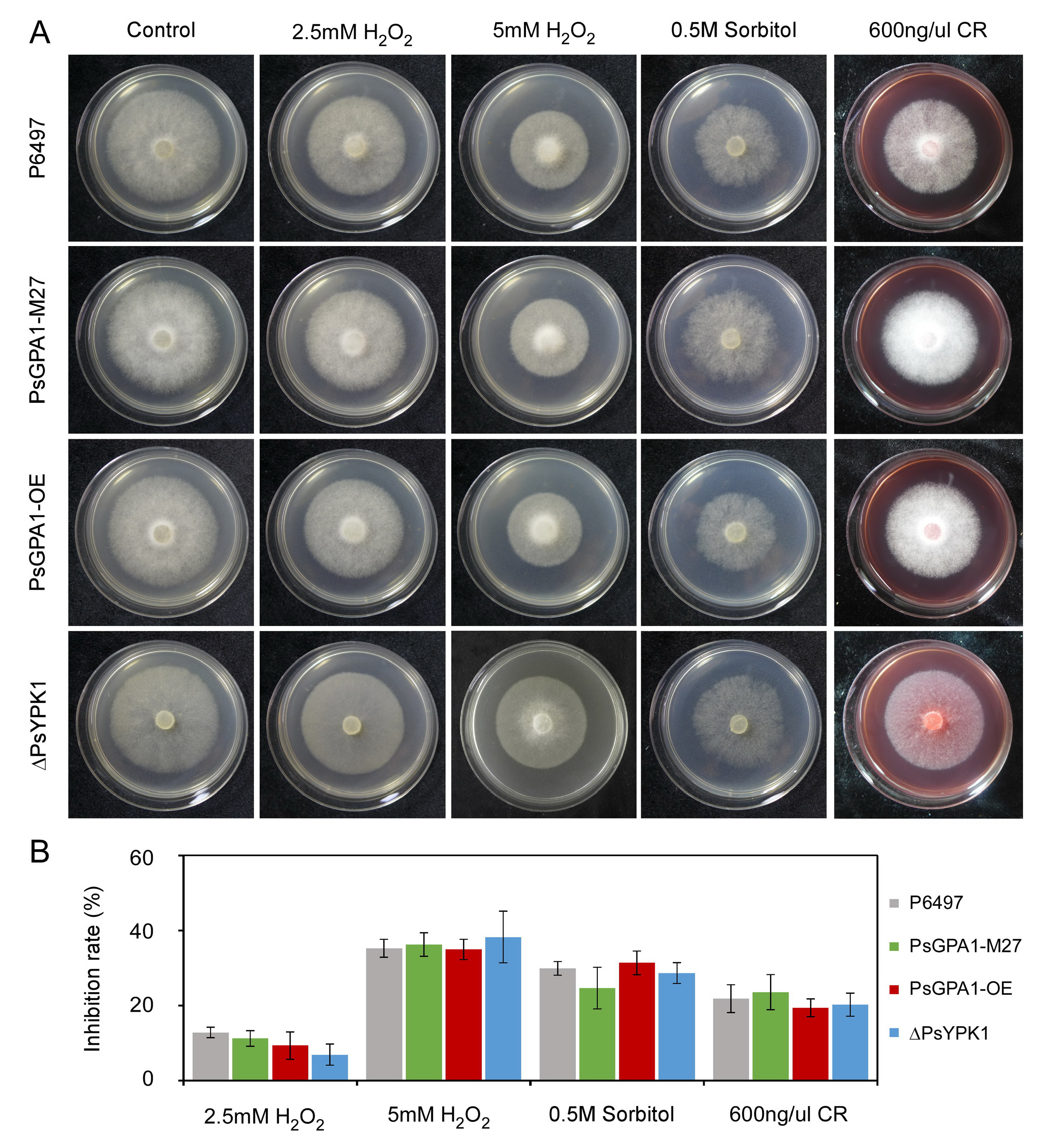

Supplement: S10 Fig — (A) Growth characteristics of wild-type (P6497), PsGPA1 silenced mutant (PsGPA1-M27), PsGPA1 overexpression strain (PsGPA1-OE) and PsYPK1 knockout mutant (ΔPsYPK1) on V8 agar medium only or supplemented with 2.5 or 5mM H2O2, 0.5 M sorbitol or 600ng/ul Congo red (CR). (B) Colony diameters were measured in each independent biological experiment after 4 days of growth. Rates of growth inhibition were calculated for each treatment relative to growth on V8 agar medium only. (TIF) [file ppat.1008138.s010.tif]

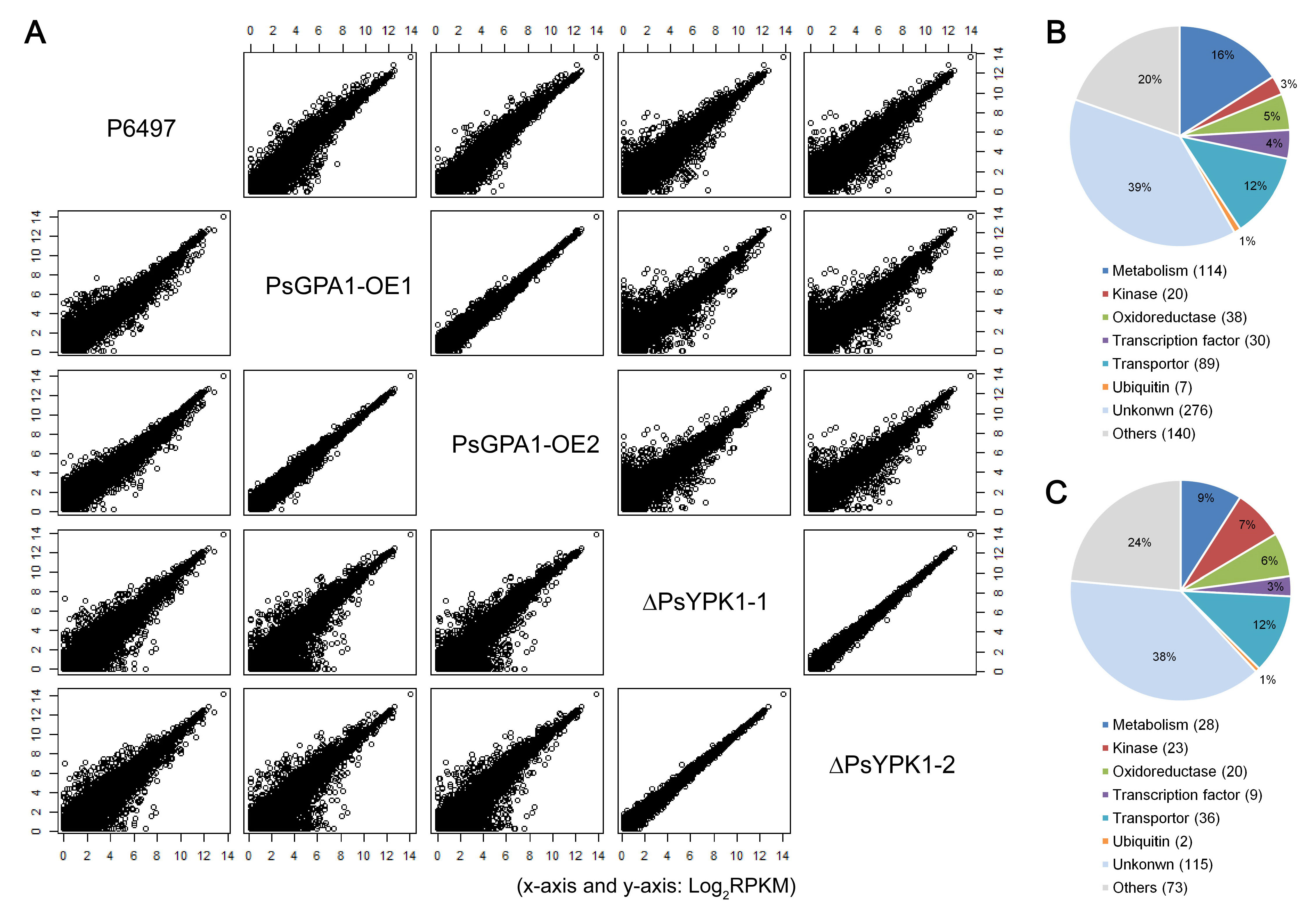

Supplement: S11 Fig — (A) Global statistical assessment of biological replicates. Scattering matrix analysis of fragments per kilobase of exon model per million mapped reads (FPKM) from all of the transcriptome samples, including the wild-type (P6497), PsGPA1 overexpression (PsGPA1-OE1 and PsGPA1-OE2), and PsYPK1 knockout (ΔPsYPK-1 and ΔPsYPK1-2) samples. One of the P6497 samples failed in library construction, so the global statistical assessment data of the P6497 biological duplicate is absent. PsGPA1-OE1 and PsGPA1-OE2, and ΔPsYPK-1 and ΔPsYPK1-2 are biological duplicates. Variation analysis was conducted not only in independent biological replicates but also between different strains. (B, C) Functional annotation and classification of specific DEGs in the PsYPK1 knockout (B) and PsGPA1 overexpression (C) samples. (TIF) [file ppat.1008138.s011.tif]

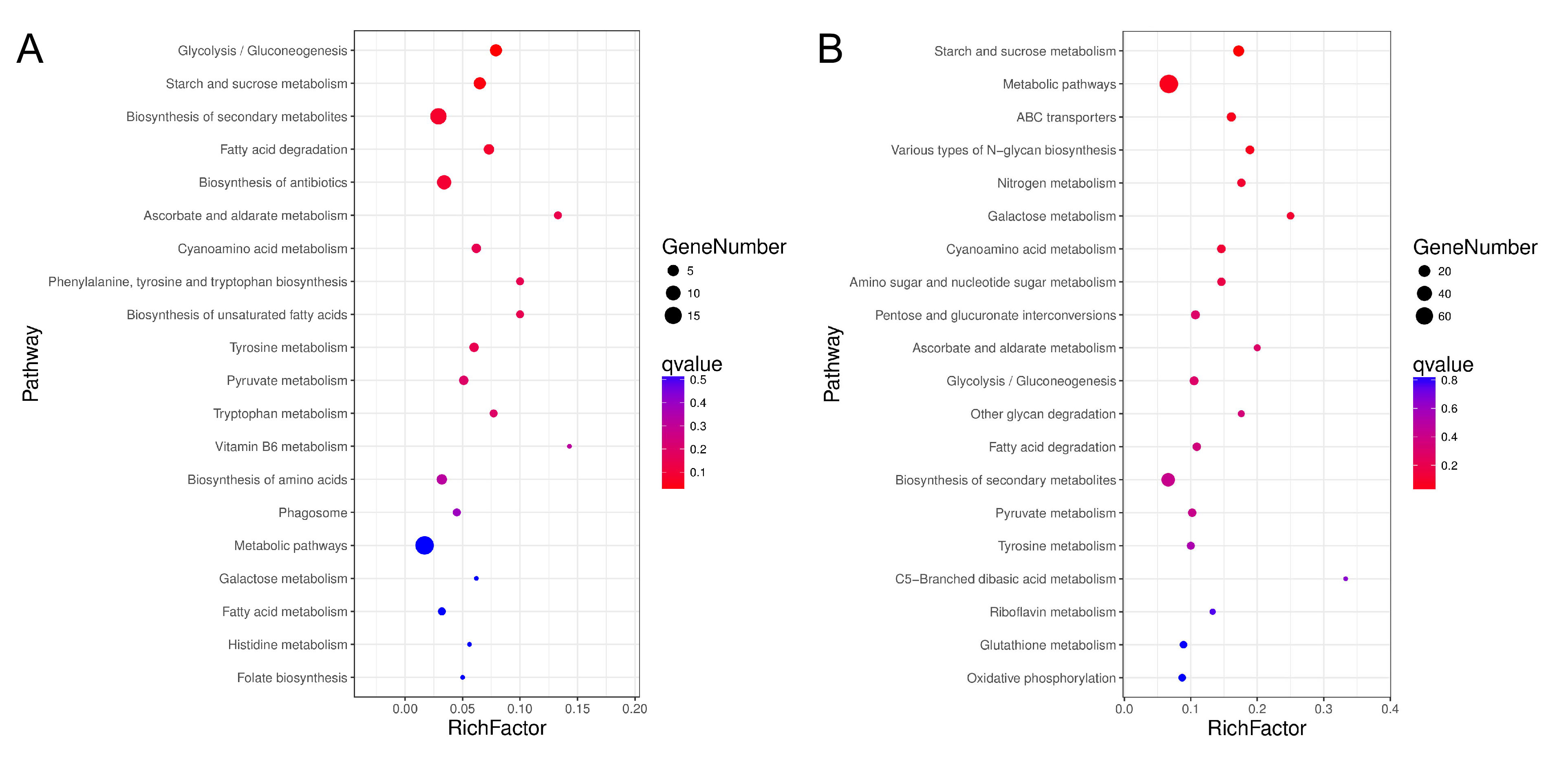

Supplement: S12 Fig — (A, B) The top 20 pathways enriched by overlapping DEGs (A) or DEGs from ΔPsYPK1 mutant (B) are displayed in an enriched scatter diagram. The rich factor, number of genes, and q value were used to measure the degree of KEGG enrichment. When a pathway with greater rich factor, larger number of genes and, q value is less than 0.05, the enrichment is more significant. (TIF) [file ppat.1008138.s012.tif]
